# Heavy boron isotopes reveal deep carbon recycling in hot subduction zones

**DOI:** 10.1038/s41467-026-78096-6

**Published:** 2026-09-24

**Authors:** Hai-Quan Liu, Feng Tian, Miao-Hong He, Ze-Xian Cui, Le Zhang, Peng-Li He, Lu-Bing Hong, Xiao-Long Huang, Ilya Bindeman, Kaj Hoernle, Yi-Gang Xu

**Affiliations:** 1 State Key Laboratory of Deep Earth Processes and Resources, Guangzhou Institute of Geochemistry, Chinese Academy of Sciences, Guangzhou, China; 2 Guangdong research center for strategic metals and green utilization, Guangzhou, China; 3 Southern Marine Science and Engineering Guangdong Laboratory (Guangzhou), Guangzhou, China; 4 School of Earth Sciences, Guilin University of Technology, Guilin, China; 5 Department of Earth Sciences, University of Oregon, Eugene, OR USA; 6 GEOMAR Helmholtz Centre for Ocean Research Kiel, Wischhofstraße 1-3, Kiel, Germany; 7 Institute of Geosciences, Ludewig-Meyn-Straße 10, University of Kiel, Kiel, Germany

**Keywords:** Geochemistry, Precambrian geology

## Abstract

The efficiency of deep carbon recycling in hot subduction systems remains debated, with contrasting models predicting either extensive shallow decarbonation or substantial carbonate retention within the subducted slab. Here we present boron-isotope and geochemical data from olivine-hosted melt inclusions in volcanic arc adakites from western Panama and in forearc high-Mg andesites from SW Japan, representing modern and Archean-like hot subduction systems. Olivine-hosted melt inclusions exhibit elevated δ^11^B values (up to +23.2‰) coupled with high Ce/Pb, La/Yb, Eu/Ti, and Th/Zr ratios, indicating melt contributions from subducted carbonate-bearing lithologies. Integration with regional CO_2_ flux estimates from western Panama suggests that more than ~60% of slab carbonate escapes shallow decarbonation, likely owing to extensive forearc and arc dehydration causing substantial fluid loss, thereby suppressing fluid-mediated decarbonation beneath both the deeper forearc and arc front. These results demonstrate that substantial carbon can bypass shallow decarbonation in both modern and Archean hot subduction systems.

## Abstract (web summary)

Carbon can survive shallow loss in hot subduction zones and reach the deep mantle in both modern and Archean subduction systems, according to isotope and geochemical data from rock-samples in western Panama and SW Japan

## Introduction

Subduction of carbonated slab transfers surface carbon into the mantle and modulates atmospheric CO_2_, underpinning Earth’s long-term carbon cycle and climate stability^1–3^. Yet the efficiency of this recycling remains hotly debated, particularly in hot subduction systems. Whereas most models predict relatively efficient carbon retention in relatively cold slabs^4–6^, contrasting predictions have emerged for hotter slabs. High-pressure-temperature experiments^7–9^ and phase equilibria calculations^4^ suggest that substantial carbonate may remain stable and survive devolatilization even along relatively warm slab geotherms^8^. By contrast, models involving externally derived fluids and flux-based estimates argue that most slab carbon is released at shallow depths—particularly in hot subduction zones (>330–360 °C GPa^−1^), where models predict near-complete decarbonation before the slab reaches sub-arc conditions^4–6,10^. In particular, given the estimated high carbon cycling efficiency of hot subduction systems (e.g., ~100% for Cascadia and ~92% for south Chile), metamorphic reactions and dissolution processes at forearc depths have been proposed to dominate decarbonation in subduction zones^5,11^. However, an independent line of evidence comes from carbonatitic signatures in intraplate magmas derived from recycled mantle domains, including carbonatites, oceanic island basalts (OIB), and OIB-type continental basalts, many with Archean–Proterozoic residence times when slab thermal gradients were likely higher (e.g., >400 °C GPa^−1^)^6^, indicates that substantial carbonate may have bypassed shallow decarbonation and been transported into the deep mantle^12–16^.

Resolving this discrepancy requires direct constraints from slab-derived melts in volcanic arc settings (forearc, volcanic front, and rear arc), where carbonate either undergoes complete decarbonation or partially escapes it. Beneath arcs, near-isobaric heating of the down-going slab drives extensive breakdown of hydrous minerals, which can promote efficient decarbonation. Therefore, the sub-arc mantle is a major locus of slab carbon loss following fore-arc devolatilization^5,11,17^. At greater depths beyond the arc, adiabatic compression stabilizes prograde hydrous phases, markedly suppressing fluid release^18^ and thereby inhibiting fluid-mediated decarbonation. Despite this framework, the fate of carbon during slab melting in hot subduction zones remains poorly constrained by direct geological evidence, limiting our understanding of decarbonation behavior in both modern and ancient high-thermal-gradient subduction systems.

**Fig. 1:**
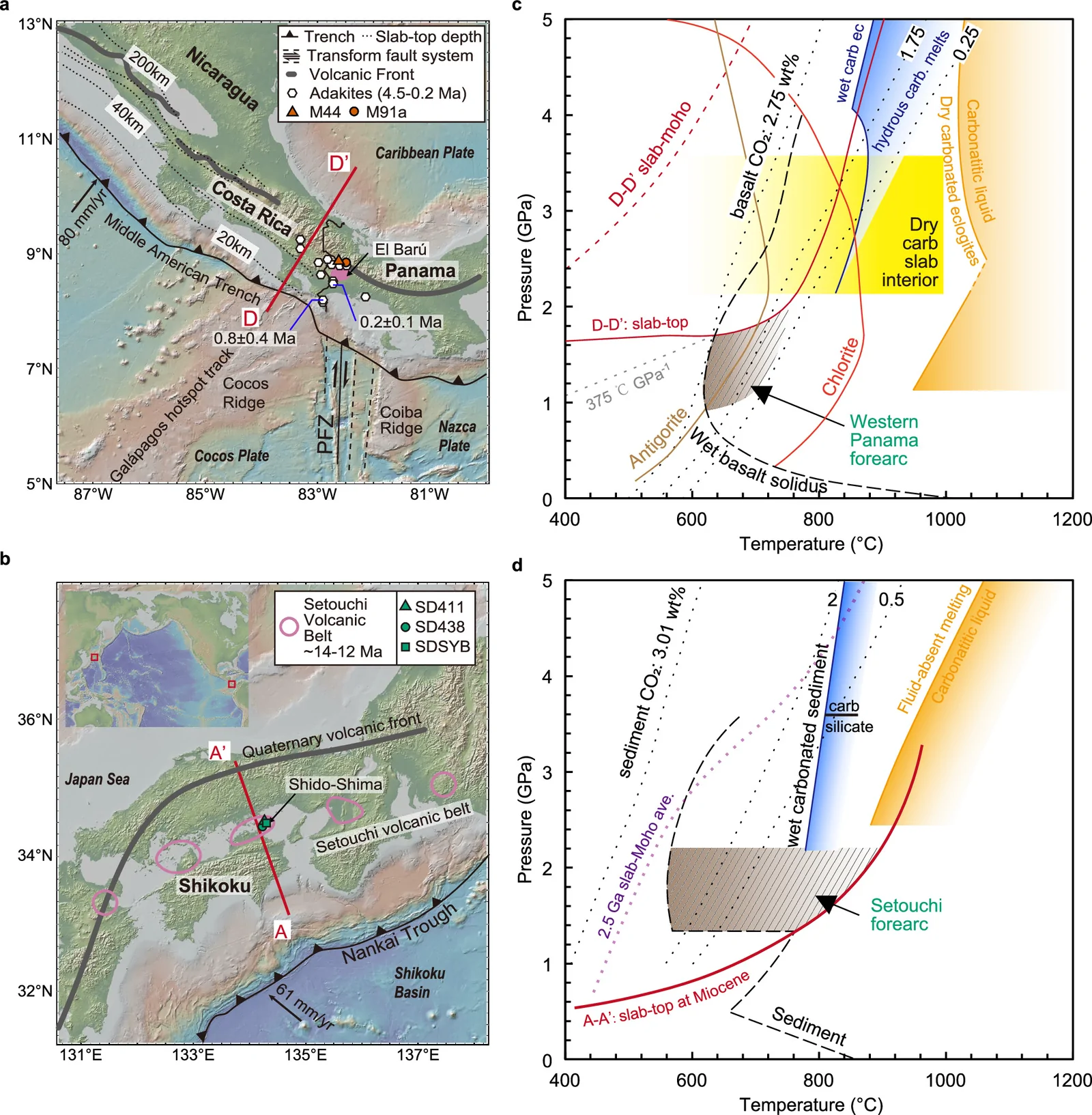
Tectonic framework and slab thermal structure of the southern Central American Volcanic Arc and the Setouchi Volcanic Belt. **a**, **b** Regional tectonic elements and the distribution of adakites (empty hexagons and orange triangle and circle) and high-Mg andesites (green triangle, circle, and square) are based on refs. ^20–22^. The base maps were generated using GeoMapApp (www.geomapapp.org) with the Global Multi-Resolution Topography (GMRT) synthesis^61^, licensed under CC BY 4.0 (https://creativecommons.org/licenses/by/4.0/)/ CC BY / CC BY. **c**, **d** The occurrence of adakites and high-Mg andesites in forearc regions indicates elevated slab-top geotherms that intersect the wet basalt^55^ and sediment^20^ solidi (dashed black lines), inducing melting of the subducted slab at forearc depths (hatched fields). The slab-top (burgundy curve) and Moho (dashed burgundy curve) geotherms^46^ of the southern Central American Volcanic Arc do not intersect the solidi of dry (orange curve)^9^ and hydrous (dark blue curve)^3,17^ carbonated eclogites, respectively. Solidi of antigorite (brown curve) and chlorite (reddish orange curve) are based on refs. ^62,63^. The gradational yellow shading indicates the inferred geotherms of the subducted slab interior beneath western Panama. The Miocene slab-top geotherm of the Setouchi Volcanic Belt^20^ intersects both the solidi of wet carbonated sediments and the fluid-absent melting curve of carbonated sediments^20,64,65^. Archean slab-Moho geotherm (dotted purple line) was extrapolated to 2.5 Ga from the average conditions of modern subduction zones^66^, assuming a secular mantle cooling rate of about 70 °C Gyr^−1^^3,67^. Data supporting this figure are provided in Supplementary Data 1. Dotted lines with numbers denote metamorphic devolatilization of subducted crust^4,68^, where decreasing values indicate greater decarbonation. Brown, blue, and orange shaded fields denote silicate melts, hydrous carbonatitic melts, and anhydrous carbonatitic melts, respectively. Abbreviations: PFZ Panama Fracture Zone; carb carbonatitic/carbonated; ec eclogites.

Both the southern Central American Volcanic Arc (CAVA) in southern Costa Rica and Panama, and the Setouchi Volcanic Belt (SVB) in SW Japan represent rare modern and ancient examples of hot subduction systems^19,20^. Widespread forearc, arc front, and rear arc adakites^21–23^ in southern CAVA document slab-edge melting at an exceptionally high thermal gradient (>~375 °C GPa^−1^) (Fig. 1), driven by slab-window formation and thermal input from the Galápagos plume^21–23^. The SVB high-Mg andesites (HMA), emplaced trenchward (in the forearc) of the Quaternary volcanic front (Fig. 1), exhibit Hf isotope compositions that point to a sedimentary contribution, revealing partial melting of subducted sediments^20^. Given the ubiquity of carbonate in the subducting slab, including altered oceanic crust (AOC)^12^, sediments^18,24^, and serpentinized lithospheric mantle^25^, adakites and HMAs from these two subduction systems plausibly sample carbonate-bearing slab components. Because slab melts are typically characterized by light boron isotopic compositions owing to forearc dehydration^26–28^, anomalously heavy boron combined with incompatible-element ratios in olivine-hosted melt inclusions could provide a diagnostic tracer of subducted carbonate that bypassed shallow isotopic fractionation and retained its seawater-derived signature^13^.

Here we present boron-isotope and incompatible-element data for olivine-hosted melt inclusions (MIs) from Quaternary arc adakites (volcanic front: M91aKH; rear arc: M44KH) near El Barú volcano in western Panama^21,22,29^ and those of Miocene forearc Shido-Shima basalt (SDSYB) and high-Mg andesites (SD411 and SD438; hereafter termed high-Mg andesites together) in SW Japan (Fig. 1)^20^. The results show that, as MI δ^11^B increases from −13.4‰ to +23.2‰, Ce/Pb (94–361), La/Yb (27–165), Eu/Ti (0.00030–0.00036), and Th/Zr (0.043–0.056) ratios also increase, indicating slab melting in both forearc and sub-arc settings as a key mechanism for decarbonation. These observations, together with the low carbon cycling efficiency inferred for western Panama (~11–38%), suggest that extensive early fluid loss in hot subduction zones suppresses subsequent fluid-mediated decarbonation processes, including metamorphic reactions, carbonate dissolution, and melting, both beneath the deeper forearc and within the sub-arc region.

## Results

### Geochemistry of Panama and Shido-Shima melt inclusions

**Fig. 2:**
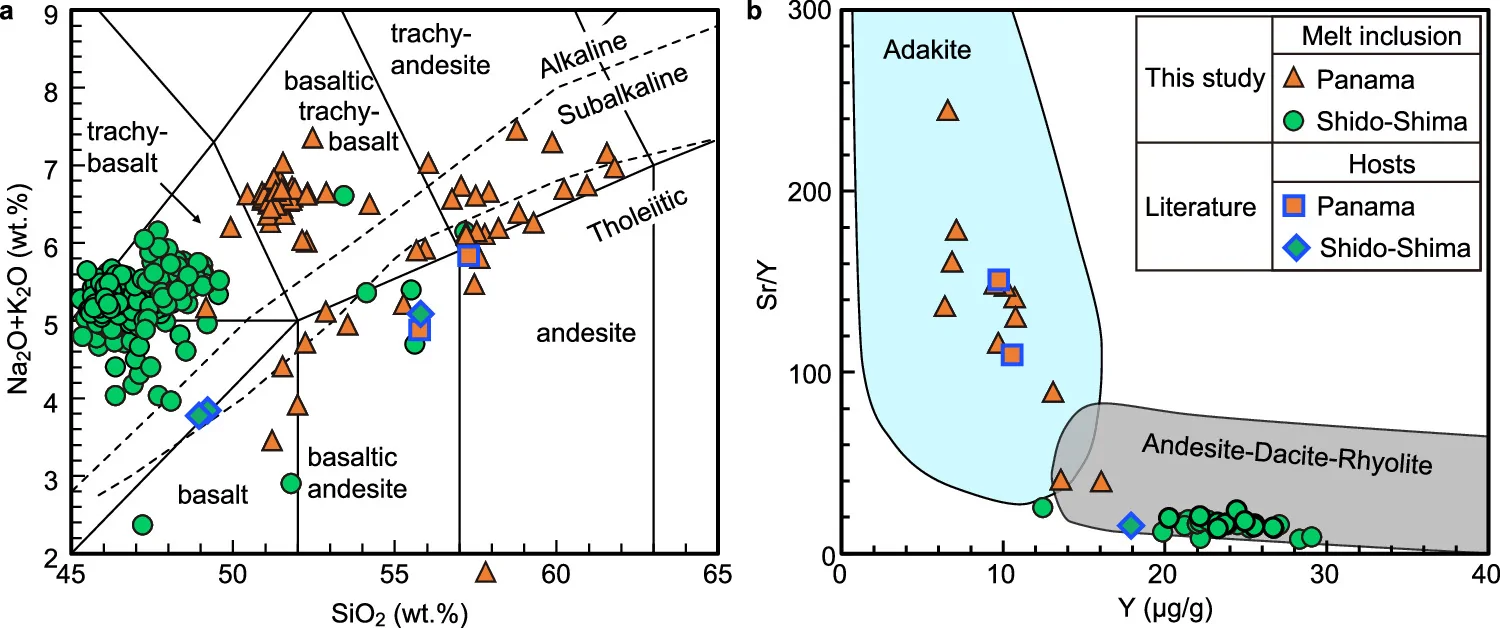
Plots of total alkalies versus SiO_2_ (wt.%) and Sr/Y versus Y (μg/g) for Panamanian and Shido-Shima melt inclusions. **a** Panamanian melt inclusions (orange triangles) display a broader range of SiO_2_ and higher total alkalies than their host adakites (orange squares with blue margins), whereas Shido-Shima melt inclusions (green circles) are characterized by lower SiO_2_ but higher total alkalies relative to their host high-Mg andesites (green diamonds with blue margins). **b** Compared with Shido-Shima melt inclusions, those from Panama display higher Sr/Y ratios. The discriminant fields for rock types (cyan, adakites; gray, andesite-dacite-rhyolite suite magmas) are based on refs. ^19,69^.; bulk-rock major and trace element compositions are from refs. ^29,58,70^. Data supporting this figure are provided in Supplementary Data 1.

**Fig. 3:**
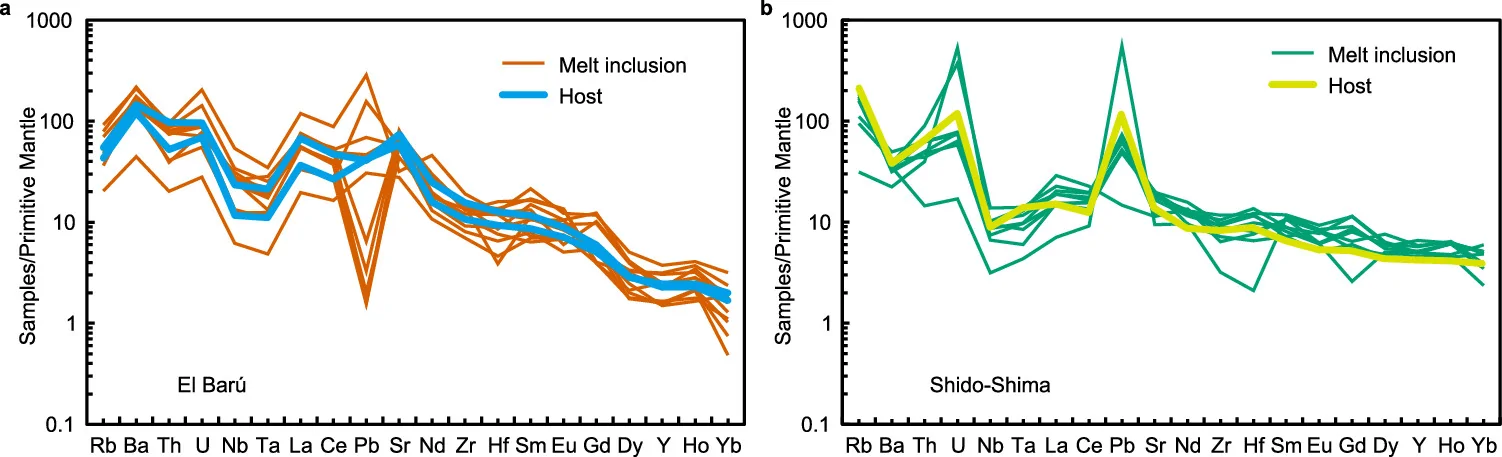
Comparison of primitive mantle^71^-normalized trace element diagrams for Panamanian and Shido-Shima melt inclusions and their host lavas^70^. **a** Panamanian melt inclusions (orange lines) exhibit steeper right-sloping trace element patterns similar to their host adakites (blue lines), except that the former exhibit positive and negative Pb anomalies and two samples show negative Hf anomalies. **b** Shido-Shima melt inclusions (green lines) exhibit relatively flat trace element patterns broadly similar to that of the host high-Mg andesite (yellow-green line), with the exception that one sample shows no Pb anomaly. Data supporting this figure are provided in Supplementary Data 1.

**Fig. 4:**
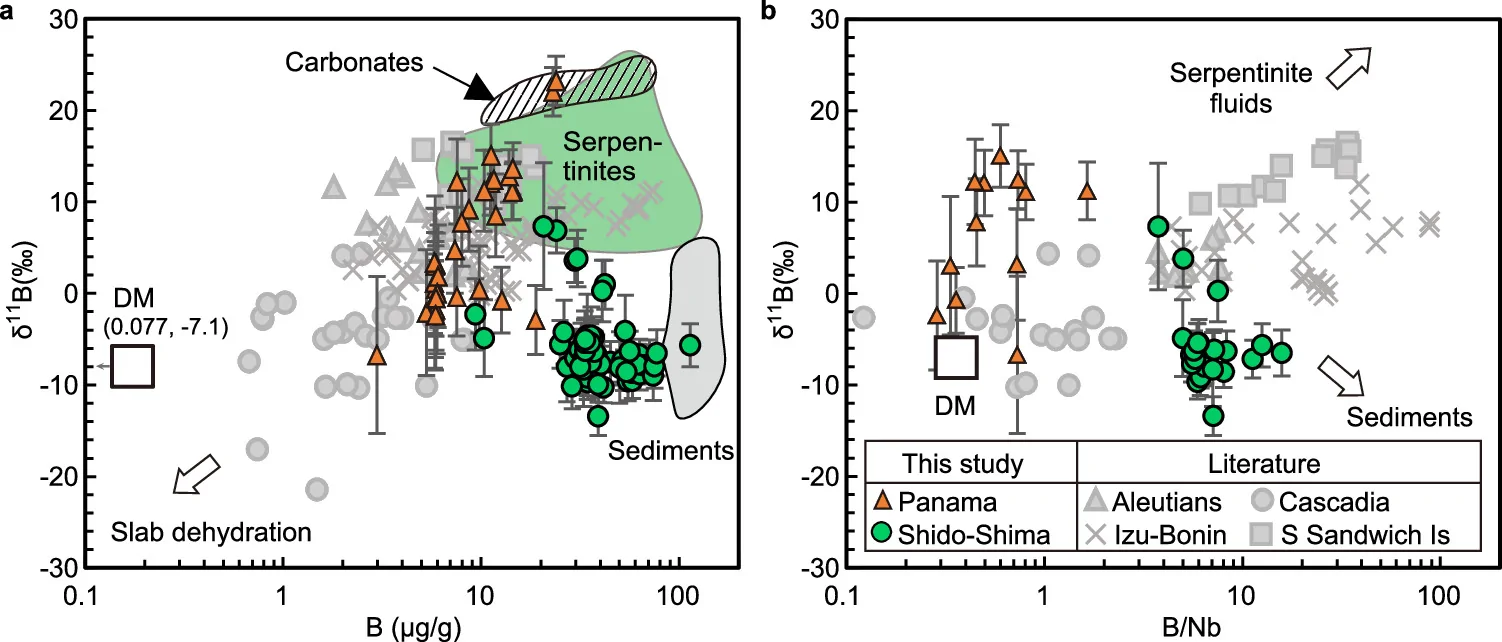
Plots of δ^11^B versus Boron (μg/g) and B/Nb for Panamanian and Shido-Shima melt inclusions. **a** Panamanian melt inclusions (orange triangles) generally exhibit enriched boron isotope compositions and moderate B concentrations, plotting close to the serpentinite^36,72^ (green area) and carbonate^39^ (hatched area) fields in terms of δ^11^B and B contents. In contrast, Shido-Shima melt inclusions (green circles) are essentially characterized by light boron isotope compositions and high B concentrations, approaching the pelagic sediments^34^ (gray field). **b** A subset of isotopically heavy Shido-Shima melt inclusions falls within the field defined by serpentinite fluid-influenced magmas from the Aleutian^35^ (gray triangles), Izu-Bonin^33,73^ (gray crosses), and South Sandwich^36^ (gray rectangles) subduction systems, whereas Panamanian MIs define a steeper trend with a slope exceeding those of published arc magma arrays. Data for depleted mantle (DM, empty squares), pelagic sediments, and Cascadia (gray circles) are from refs. ^26,27,34,48,74–76^. Error bars represent 2SE. Data supporting this figure are provided in Supplementary Data 1.

**Fig. 5:**
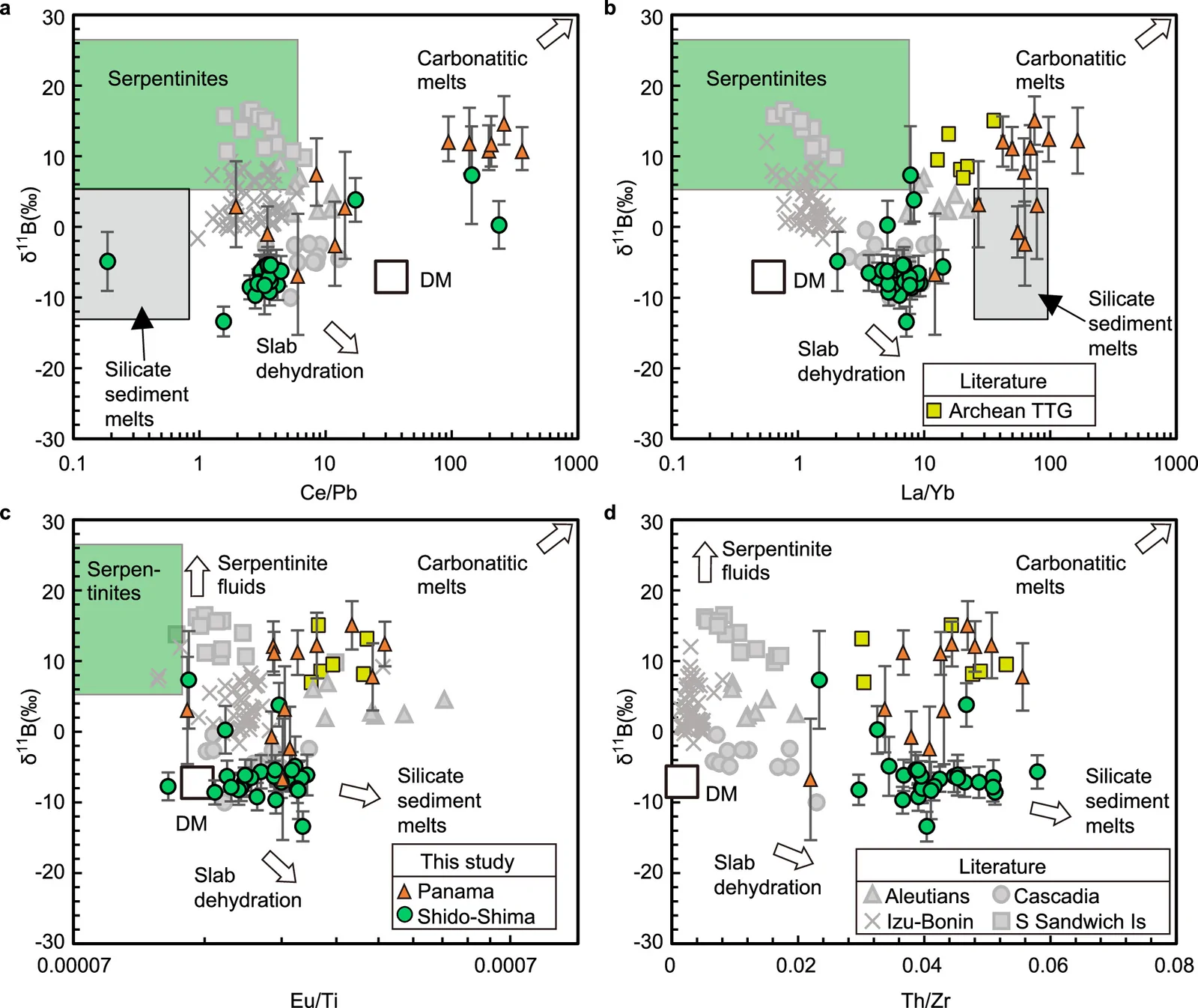
Boron isotopic enrichment associated with variations of carbonatitic proxies of Panamanian and Shido-Shima melt inclusions. **a**–**d** Compared with mantle wedge serpentinites (green fields)^31,72^ and normal arc lavas (gray rectangles: South Sandwich Islands^36^; gray crosses: Izu–Bonin^33,73^) that typically reflect serpentinite-fluid contributions, a subset of heavy-B Panamanian (orange triangles) and Shido-Shima (green circles) melt inclusions identified in this study, together with isotopically heavy Archean tonalite–trondhjemite–granodiorite suites (TTG; yellow-green squares)^52^, exhibit elevated carbonatitic proxies. Some Aleutian^35^ (gray triangles) and Cascadia^26,48,75,76^ (gray circles) samples may also reflect contributions from carbonate-, serpentinite-, and silicate sediment-derived components in their sources. Depleted mantle (DM, empty squares) is based on refs. ^27,74^. Silicate sediment melt compositions (gray fields) are based on refs. ^34,77^. Error bars represent 2SE. Data supporting this figure are provided in Supplementary Data 1.

Olivine-hosted MIs from adakites in Panama near El Barú display a wide range of major element compositions (SiO_2_ = 49.2–61.8 wt.%; total alkalies = 3.5–7.3 wt.%; Supplementary Data 2) and are generally characterized by elevated Sr/Y ratios (>40; Fig. 2). Their trace element patterns show enrichment in incompatible elements and are broadly similar to those of the host rocks, although they exhibit variable Pb anomalies and two samples show negative Hf anomalies (Fig. 3). Compared with their host HMAs, Shido-Shima MIs contain lower SiO_2_ contents (45.3–57.2 wt.%) but higher total alkalies (2.4–6.6 wt.%), and display lower Sr/Y ratios than the Panamanian MIs (Fig. 2). Their trace element patterns are broadly comparable to those of the host HMAs, except for one sample without a Pb anomaly and minor departures of several elements from the main array (Fig. 3). Together, Panamanian and Shido-Shima MIs span a wide range of δ^11^B ([(^11^B/^10^B)_sample_/(^11^B/^10^B)_NISTSRM951_ − 1] × 1000 = −13.4 ± 2.1‰ to +23.2 ± 2.7‰) isotope compositions and concentrations (3.0–113.9 μg/g) between the fields of depleted mantle, sediments, serpentinites, and carbonates (Fig. 4). Notably, a subset of MIs with heavier B isotope compositions are associated with elevated Ce/Pb (94–361), La/Yb (27–165), Eu/Ti (0.00030–0.00036), and Th/Zr (0.043–0.056) ratios, deviate from the serpentinite trends and extend towards trends of carbonatitic melts (Fig. 5).

Although upper-plate magmatic differentiation can modify whole-rock compositions, olivine crystallizes early in basaltic to andesitic systems^30^, such that olivine MIs largely retain primary melt signatures. This inference is supported by the predominantly primitive nature of the MIs, with 308 of 346 inclusions containing ≤ 52 wt.% SiO_2_, in contrast to the more differentiated host HMAs (49.2–55.8 wt.%) and adakites (55.8–57.3 wt.%; Fig. 2a and Supplementary Data 2). Moreover, MI δ^11^B and B/Nb, Ce/Pb, La/Yb, Eu/Ti, and Th/Zr ratios show no systematic relationship with olivine forsterite content (Fo = Mg/(Fe + Mg)) (Supplementary Fig. 1), indicating that these geochemical characteristics primarily reflect mantle source signatures rather than upper-plate degassing or differentiation processes.

### Light boron signatures in melt inclusions

In SW Japan and western Panama, the host lavas of the olivine MIs have radiogenic isotopic signatures indicative of subducted sediments and hotspot tracks, respectively, and are therefore interpreted as products of interaction between slab-derived melts and the mantle wedge^20–22^. Given that boron undergoes strong isotopic fractionation during slab dehydration and melting^27,28,31,32^, the low δ^11^B values (−13.4 ± 2.1‰ to −2.9 ± 3.8‰) with trace element ratios approaching the depleted mantle (Figs. 4 and 5) observed in the Shido-Shima and Panamanian MIs can be readily explained by combined contributions from the mantle wedge and subducted oceanic components (AOC and silicate sediments). In addition to the slab dehydration, we infer contributions from the unmodified mantle wedge and isotopically unfractionated silicate sediments to the overall light B signature. The basaltic–andesitic SiO_2_ contents of the MIs are consistent with partial melts of peridotite or reaction pyroxenites^20,21^, suggesting that part of their light B signature reflects input from the ambient depleted mantle wedge (δ^11^B = −7.1 ± 0.9‰)^27^ (Fig. 4). Moreover, because sedimentary B is predominantly hosted in phengite, which remains stable to relatively high temperatures (>800 °C)^18^, light B from subducted sediments can be transported to sub-arc depths with minimal isotopic fractionation during slab dehydration.

### Heavy boron signatures in melt inclusions

The studied MIs (Panama: M91aKH-8, M91aKH-32, M91aKH-46, and M44KH-8; Shido-Shima: SD411-1, SD411-27, SDSYB-16, and SDSYB-27) with moderate δ^11^B values (−0.7 ± 3.6‰ to +7.8 ± 4.8‰; Supplementary Data 2 and Fig. 5) may record a contribution from serpentinite-derived fluids. Their boron isotope compositions and trace element ratios (Ce/Pb, La/Yb, and Eu/Ti) are comparable to those of arc magmas from the South Sandwich, Izu–Bonin, and Aleutian systems (Fig. 5), where serpentinite-derived fluids are believed to play a significant role in magma genesis^33–36^. Because Pb and B are fluid-mobile, whereas light rare earth elements (LREEs) are relatively immobile, serpentinization of the forearc mantle by slab-derived fluids is expected to produce higher δ^11^B and lower Ce/Pb relative to depleted mantle, while La/Yb and Eu/Ti remain largely mantle-like. Subsequent dehydration or partial melting of serpentinized forearc mantle that is dragged down to deeper depths may transfer these signatures into forearc and arc magmas^34,37,38^. However, serpentinite-mediated slab fluids cannot account for a subset of Shido-Shima and Panamanian MIs characterized by moderate to high δ^11^B values (+0.3 ± 3.4‰ to +23.2 ± 2.7‰) together with high Ce/Pb (up to 361), La/Yb (165), Eu/Ti (0.00036) and Th/Zr (0.056) (Fig. 5). The absence of covariation between δ^11^B and serpentinite-sensitive tracers such as (As, Sb)/Ce (Supplementary Fig. 2) further argues against serpentinite fluids as the dominant control on these compositions.

Carbonates formed in seawater environments commonly exhibit heavy B isotope compositions^39^ (Fig. 4a), suggesting that the boron isotopic enrichment in the Panamanian and Shido-Shima MIs may instead reflect contributions from subducted carbonate-bearing components, including carbonated AOC and sediments. Moreover, carbonates may precipitate within the subducting slab lithospheric mantle as ophicarbonates, formed through interactions between serpentinite and seawater-related hydrothermal fluids at mid-ocean ridges or along bend faults^25^, thereby inheriting the heavy boron isotopic signature of seawater (+39.6‰)^40^. The elevated Ce/Pb, La/Yb, Eu/Ti, and Th/Zr ratios may arise from elemental fractionation during partial melting of carbonates. Carbonatitic melts can efficiently scavenge light and middle REEs and Th released during the breakdown of accessory phases such as phosphates and allanite, given these elements are strongly compatible in carbonatitic melts relative to heavy rare earth elements (e.g., Yb) and high field strength elements (e.g., Ti and Zr)^12,41^. Moreover, experimental work shows that carbonates have D^solid/melt^_Ce/Pb_ and D^solid/melt^_La/Yb _< 1^42^, which would lead to the resulting carbonatitic melts having elevated Ce/Pb and La/Yb ratios. Collectively, these isotopic-elemental systematics indicate that the moderate to high δ^11^B values recorded by the Shido-Shima and Panamanian MIs reflect contributions from carbonatitic melts rather than serpentinite-derived fluids.

## Discussion

Given that slab-derived adakites have been reported from the forearc regions of both western Panama^22^ and Cascadia (e.g., Mount St. Helens)^19,43^, western Panama is inferred to have slab thermal gradients comparable to, or higher than, those of Cascadia (Supplementary Fig. 3). If decarbonation of the Cocos ridge were completed at forearc depths through metamorphic reactions and carbonate dissolution, arc magmas would not be expected to preserve substantial slab-derived carbon signatures. Instead, the geochemical characteristics of the Panamanian MIs indicate that slab carbon was transferred into the sub-arc mantle. In particular, carbon flux estimates^1,24,25,44^ that account for carbon supplied by slab serpentinites suggest a shallow arc-system carbon cycling efficiency of 11–38% (Supplementary Data 3). One proposed explanation is that carbon lost from the slab was sequestered in the overriding-plate lithospheric mantle through fluid–mantle interaction and formation of listvenites (magnesite/dolomite + quartz)^10^. Such carbon sequestration may be particularly efficient in the forearc mantle wedge, where extensive serpentinization provides abundant reactive peridotite for carbon storage. However, seismic observations reveal no identifiable serpentinized peridotite within the sub-arc lithospheric mantle^45^, suggesting a substantially lower capacity for carbon sequestration beneath the arc than in the forearc mantle wedge. We therefore infer that the “missing” carbon in the southern CAVA is more likely transported beyond sub-arc depths within the subducting slab lithosphere, rather than being retained within the sub-arc lithospheric mantle.

The efficiency of metamorphic decarbonation and carbonate dissolution is ultimately governed by fluid availability and the physicochemical properties of slab-derived fluids, including the stability of dissolved ionic complexes^5,11^. Under fluid-limited conditions, both metamorphic reactions and carbonate dissolution are expected to be strongly suppressed. In western Panama, elevated forearc slab-top temperatures exceed the dehydration solidi of major hydrous minerals within the oceanic crust, including lawsonite and phengite (Supplementary Fig. 3). This, together with the eclogitic melt-like geochemical signatures of the forearc adakites^22^ that exclude significant amphibole involvement, indicates that hydrous minerals in the sub-arc oceanic crust are unlikely to supply substantial fluids. Slab–Moho temperatures beneath southern Costa Rica^46^, located ~80 km northwest of western Panama, remain within the stability field of antigorite and chlorite (Fig. 1c), potentially allowing the serpentinized lithospheric mantle of the down-going slab to supply additional fluid for decarbonation of the overlying oceanic crust. However, thermal modification by the Galápagos plume^22,23^ may have altered slab thermal conditions beneath western Panama. Specifically, plume-related heating may have elevated slab temperatures and promoted earlier dehydration and fluid loss from the slab mantle at shallower forearc depths, thereby reducing fluid transfer to greater depths. This interpretation is consistent with the observation that only a small proportion (possibly 3 out of 12; Fig. 5a) of Panamanian MIs show signatures indicative of a possible contribution from serpentinite fluids. However, these signatures are not uniquely diagnostic of serpentinites, as mixing between silicate sediment- and carbonate-derived melts may provide an alternative explanation. Thus, a minor serpentinite contribution cannot be excluded, but it is unlikely to account for the dominant heavy B isotopic signatures in the Panamanian MIs. More broadly, the link between heavy B signatures and carbon cycling is further supported by their coupled southward decline in arc lavas along the CAVA^44,47^. Lower slab temperatures beneath northern CAVA shift dehydration reactions to greater depths and favor fluid delivery into sub-arc regions, whereas warmer and shallower subduction beneath southern CAVA promotes fluid exhaustion before the slab reaches the forearc magma generation zone^44,47^. We therefore propose that heating by the Galápagos mantle plume promoted rapid and early dehydration of the subducting slab at shallow forearc depths. As a result, by the time the slab reached greater depths, although slab temperatures had increased, they remained below the solidus of dry carbonated eclogites (Fig. 1c), thereby preventing extensive carbonate melting and fluid-assisted decarbonation.

Although heavy B isotope compositions in adakites from the Aleutian and Cascadia subduction systems have previously been attributed to contributions from serpentinite and/or AOC^35,48^, Fig. 5 shows that a subset of the Aleutian samples also exhibit some elevated incompatible element ratios such as La/Yb and Eu/Ti that overlap or approach the trend defined by the Panamanian MIs. This suggests that part of the slab-derived carbon of this subduction system may have also escaped shallow decarbonation. Estimates that integrate carbon inputs from subducted sediments^49^, altered oceanic crust^24^ and serpentinized mantle^25^ with carbon emissions from forearc and arc volcanism^43,50,51^ suggest that Cascadia also has a shallow carbon cycling efficiency of ~24% (Supplementary Data 4), despite there being no clear carbonate signature in the geochemistry of these lavas in Fig. 5. We therefore infer that carbonates hosted within the relatively dry oceanic crust (Fig. 1 and Supplementary Fig. 3) can, at least locally, bypass forearc decarbonation and be transported to sub-arc depths and potentially into the deeper mantle. Such a process may have also operated in Archean subduction systems (≥2.5 Ga; Fig. 1d), as indicated by 2.7–2.6 Ga tonalite–trondhjemite–granodiorite suites from northeastern China^52^ that display heavy B isotope compositions and elevated carbonatitic geochemical proxies (Fig. 5). It may also explain why some modern HIMU-type basalts carrying carbonatitic signatures preserve mantle residence ages traceable to the Archean (e.g., Mangaia)^53^, pointing to long-term storage of recycled carbonates in the deep mantle through subduction.

## Methods

### Melt inclusion homogenization

Olivine-hosted melt inclusions (Supplementary Fig. 4) were subjected to heating homogenization through melting and quenching, by using a 1-atm CO_2_-H_2_ gas mixing furnace buffered at the quartz-fayalite-magnetite (QFM) oxygen fugacity^54^ at the State Key Laboratory of Deep Earth Processes and Resources, Guangzhou Institute of Geochemistry, Chinese Academy of Sciences (DEEPER, GIGCAS). Olivine grains from two adakites (M44KH and M91aKH) and three high-Mg andesites (SD411, SD438, and SDSYB) were separately loaded into a platinum crucible that was gradually lowered, over 5 min, from the top of the furnace (100 °C) to the center of the furnace tube (1250 °C) and kept at that temperature for 5 min before being quenched at room temperature.

### EPMA analysis of major element concentrations

In situ major oxides and chlorine analyses of olivine grains and melt inclusions were conducted using a Cameca SXFive FE electron probe microanalyzer (EPMA) at DEEPER, GIGCAS. Detailed experimental parameters and procedures can be found in reference ^55^. Reference materials MG-Ol, BHVO-2G, BCR-2G and T1-G were used for major element quality control, while NIST SRM 612 served as the chlorine standard. Major element compositions were processed using Petrolog3 software to account for “Fe loss/gain” effects^56^, applying the olivine-melt equilibrium model of Ford et al.^57^. with a fixed Fe^2+^/Fe^3+^ ratio of 1.63. Initial FeO^T^ contents of trapped melt inclusions were constrained by host magma values (M44KH: 6.62 wt.%; M91aKH: 5.78 wt.%; SD411: 6.15 wt.%; SD438: 8.77 wt.%; SDSYB: 9.19 wt.%) (Supplementary Data 2), calculated as Fe_2_O_3_^T^ × 0.8998 from refs. ^29,58^. The measured major element and volatile compositions of standards agreed with recommended values within analytical uncertainties (Supplementary Data 5).

### SIMS analysis of melt inclusion B isotope compositions

Boron elemental and isotopic analyses were conducted using a CAMECA IMS 1280-HR ion microprobe at DEEPER, GIGCAS. One analytical session was conducted on 6 April 2025, and the other from 11 to 15 April 2025. In the first analytical session, a ^16^O^−^ primary beam of ~3.4 nA with an impact energy of 23 keV was used to sputter secondary ions from a ~20 μm sample area (10 μm spot size + 10 μm rastering). An energy window of ±50 eV (no offset) was applied, and a 30 × 30 µm raster was used during 240 s of pre-sputtering to eliminate the gold coating and minimize surface contamination. A peak-jumping mode sequentially measures the mass of ^10^B^+^, ^11^B^+^, and ^28^Si^+^, with 40 cycles collected per analysis, resulting in a total analysis time of approximately 33 min. The mass resolution (M/ΔM) was set to ~1500 (at 10% intensity) to resolve potential isobaric interferences of ^1^H^9^Be^+^ and ^1^H^10^B^+^ on ^10^B^+^ and ^11^B^+^. Except that a ~ 4 nA ^16^O^−^ primary beam was used during the second analytical session, all other instrumental parameters were identical to those used in the first session. Detailed procedures and experimental parameters are given in He et al^59^. Calibration utilized the reference materials BIR-1G, StHs6-80-G, GOR 132-G, and GOR 128-G, with BCR-2G as a monitor standard (Supplementary Data 6). Boron concentrations were derived from a ^28^Si-normalized calibration curve, with propagated uncertainties (slope error and analytical reproducibility) reported as 2SE (Supplementary Data 6). The B isotopic compositions are reported as δ^11^B relative to the NISTSRM951 standard. The B isotopic compositions of MIs were normalized to the measured primary standard BCR-2G, which was interspersed in the analytical sequence and has a recommended δ^11^B value of −5.44 ± 0.55‰^59^ (Supplementary Data 6). Eighteen analyses of BHVO-2G yielded a mean δ^11^B value of −8.3 ± 4.8‰ (2 SD, *n* = 6) for the first session and −7.3 ± 5.0‰ (2 SD, *n* = 12) for the second session, both of which are consistent within uncertainty with the recommended values of −5.03 ± 2.6‰ (Supplementary Fig. 5 and Data 6)^59^.

### LA-ICP-MS analysis of melt inclusion trace element concentrations

In-situ trace elements were analyzed by an ELEMENT XR (Thermo Fisher Scientific) ICP-SF-MS coupled with a 193-nm (ArF) Resonetics RESOlution M-50 laser ablation system at DEEPER, GIGCAS. Laser ablation conditions were optimized as follows: beam diameter (~33 μm), repetition rate (6 Hz), and energy density (~4 J cm^−2^). Analytical procedures and data reduction methods followed Zhang et al^60^. Calibration curves were established using two USGS reference glasses (BHVO-2G and GSD-1G). To ensure data quality, BCR-2G was analyzed as a secondary standard throughout each session, yielding average concentrations that matched recommended values within analytical uncertainty (most elements showing <10% RSD; Supplementary Data 7).

## Supplementary information

Supplementary Information

Description of Additional Supplementary Files

Supplementary Data 1

Supplementary Data 2

Supplementary Data 3

Supplementary Data 4

Supplementary Data 5

Supplementary Data 6

Supplementary Data 7

Peer Review File

## Data Availability

All data supporting the findings of this study are available within the paper and its Supplementary Information. Source Data underlying the figures are provided as Supplementary Data 1.
