# Supplementary material for: Heavy boron isotopes reveal deep carbon recycling in hot subduction zones
Source: Nat Commun. 2026 Sep 24;17:11104. doi: 10.1038/s41467-026-78096-6 (PMC13696696; doi:10.1038/s41467-026-78096-6)
Supplement: Supplementary file 1 [file 41467_2026_78096_MOESM10_ESM.pdf]

nature portfolio

Peer Review File

Heavy Boron Isotopes Reveal Deep Carbon Recycling in Hot Subduction Zones

Corresponding Author: Dr Hai-Quan Liu

This file contains all reviewer reports in order by version, followed by all author rebuttals in order by version.

Version 0:

Reviewer comments:

Reviewer #1

(Remarks to the Author)
Summary and main findings of the study: This study challenges existing models that predict near-complete decarbonation of subducted slabs before reaching sub-arc depths, especially in hot subduction zones. Using boron isotopes and geochemical data from olivine- hosted melt inclusions in El Barú adakites (Panama), the authors identify signatures of subducted carbonate-derived carbon rather than fluids from serpentinite.
Elevated δ11B values and high Ce/Pb ratios indicate that carbonate-rich lithologies can survive shallow decarbonation. Combined with regional CO2 flux estimates, the results suggest that over 60% of slab carbonate is retained and transported to greater depths, likely due to forearc dehydration limiting carbon loss.
Overall, the study demonstrates that significant carbonate recycling occurs even in hot subduction zones, supporting long-term deep carbon cycling since at least the late Archean.

Strengths and limitations of the study: This study addresses an interesting topic and provides a timely and relevant contribution to our understanding of deep carbon cycling. However, the results would benefit from a more comprehensive contextualization within the framework of recent literature. In particular, key studies such as Aiuppa et al. (2019), Werner et al. (2019), Farsang et al. (2021), and Arzilli et al. (2023) should be more thoroughly integrated. The findings of Arzilli et al. (2023) on metamorphic decarbonation and Farsang et al. (2021) on carbonate dissolution are especially pertinent and should be introduced earlier in the manuscript and discussed consistently throughout.
Moreover, the results from Arzilli et al. (2023) on metamorphic decarbonation and from Farsang et al. (2021) on carbonate dissolution should be presented in the Introduction section and discussed in throughout the manuscript. Some of the results presented here appear to confirm or build upon outcomes already reported by Arzilli et al. (2023). For this reason, the authors should more clearly articulate the rationale of the study and better highlight its novel contributions.
The overall structure of the manuscript also requires improvement. At present, the distinction between the Introduction and the Results and Discussion sections is not sufficiently clear, which affects the readability of the paper. In addition, a dedicated Conclusions section is currently missing and should be included to effectively summarize the main findings and their broad implications.

Recommendation: I have provided several comments and suggestions throughout the manuscript that I hope will help strengthen the study. In my opinion, the manuscript has the potential to be suitable for publication in Nature Communications, but only after substantial revision.

Detailed Comments
- Introduction
Lines 36-39: The authors stated: “Flux-based estimates argue that most slab carbon is released at shallow depths—particularly in hot subduction zones (>330–360 °C GPa-1), where models predict near-complete decarbonation before the slab reaches sub-arc conditions.” Recent relevant articles are not considered here (Farsang et al., 2021; Arzilli et al., 2023). Particularly, recent models present a global arc-by-arc lithology-specific analysis of the magnitude of slab CO2 released purely by metamorphic decarbonation of carbonate- bearing sediment and basalt during subduction of altered oceanic crust (Arzilli et al., 2023). The authors have found that metamorphic decarbonation is highly efficient in low carbonate sediments, such as carbonated clay, and in carbonate-bearing basalts of altered oceanic crust, causing all of their CO2 to be removed (Arzilli et al., 2023). Sediments with medium and higher carbonate contents, such as chalk and limestone, are only partially

decarbonated. The warmest slabs, such as Mexico and Cascadia, produce complete metamorphic decarbonation of carbonate-poor sediments beneath fore-arcs. Under more common intermediate-warm thermal regimes, and sediments with higher carbonate contents, such as most of the East Pacific subduction zones with carbonate-rich sediments (Columbia-Ecuador, Nicaragua, Guatemala-El Salvador, Costa Rica and Peru) only partial metamorphic decarbonation of carbonate minerals occurs at depths between ~80 and 170 km (~2.3 to 5.5 GPa) (see Figure attached).

- Fate of subducting carbonates at hot subduction systems

Lines 161-195: The discussion in this section does not adequately consider key studies that have quantified global volcanic CO2 emissions (e.g., Aiuppa et al., 2019; Werner et al., 2019). Incorporating these works would provide an essential framework for interpreting the results. In addition, the manuscript does not sufficiently address the fate of subducting carbonates in hot subduction zone settings. This aspect should be better contextualized by comparing CO2 outputs from volcanic arcs (Aiuppa et al., 2019; Werner et al., 2019) with estimates of CO2 input from sub-arc slabs (Farsang et al., 2021; Arzilli et al., 2023). Such a comparison is critical to properly constrain the role of subduction processes in the deep carbon cycle and to place the study's findings into a broader geodynamic perspective.

References

Aiuppa, A., Fischer, T.P., Plank, T., Bani, P., 2019. CO2 flux emissions from the Earth's most actively degassing volcanoes, 2005–2015. Sci. Rep. 9, 1–17. https://doi.org/ 10.1038/s41598-019-41901-y.

Arzilli, F., Burton, M., La Spina, G., Macpherson, C.G., van Keken, P.E. and McCann, J., 2023. Decarbonation of subducting carbonate-bearing sediments and basalts of altered oceanic crust: Insights into recycling of CO2 through volcanic arcs. Earth and Planetary Science Letters, 602, p.117945.

Farsang, S., Louvel, M., Zhao, C., Mezouar, M., Rosa, A.D., Widmer, R.N., Feng, X., Liu, J. and Redfern, S.A., 2021. Deep carbon cycle constrained by carbonate solubility. Nature Communications, 12(1), p.4311.

Werner, C., Fischer, T.P., Aiuppa, A., Edmonds, M., Cardellini, C., Carn, S., Chiodini, G., Cottrell, E., Burton, M., Shinohara, H., Allard, P., 2019. Carbon dioxide emissions from subaerial volcanic regions: two decades in review. In: Orcutt, B.N., Daniel, I., Dasgupta, R. (Eds.), Deep Carbon Past to Present. Cambridge University Press, pp. 188–236.

Reviewer #2

(Remarks to the Author)

This study presents novel boron isotope and geochemical data from olivine-hosted melt inclusions in adakites from western Panama, providing strong evidence for efficient deep recycling of carbonates in a hot subduction zone. The manuscript is well structured, the dataset is robust, and the interpretations are generally convincing. I consider the manuscript suitable for publication after addressing the following minor comments.

I suggest moving the major and trace element figures from the Supplementary Information into the main text to improve data accessibility. In addition, Pb should be included in the trace element patterns.

It would also be valuable to further examine additional carbonatitic proxies, such as SiO2, CaO, La/Yb, Zr/Hf, and Ti/Eu, to better constrain the potential carbonatite signature in the melt inclusion compositions. Including an additional figure would further enhance clarity and readability.

If possible, please include representative petrographic images of the melt inclusions in the supplementary information. Furthermore, presenting the major and trace element compositions of the host olivine would help to better constrain the stage of magma differentiation during which the melt inclusions were trapped.

Minor comments:

Lines 39–41: please note that carbonates in intraplate magma can also be derived from the deep mantle rather than subduction.

Lines 47–48: please check the sentence: "rendering the sub-arc mantle as the final major locus of slab carbon loss following fore-arc devolatilization."

Figure 1b: this figure gives the false impression that all carbon can be subducted into the deep mantle. It is suggested to add more solidi of carbon-bearing materials in subduction zones.

Lines 72–75: it would be better to briefly describe how boron isotopes of olivine-hosted melt inclusions were measured.

Figure 2: please check the carbonatitic proxies: La/Yb, Zr/Hf, Ti/Eu.

Lines 364–368: please add a figure in the supplementary information comparing the measured B isotopes of the reference materials with their recommended values.

Supplementary information: I suggest moving the major and trace element figures from the supplementary information into the main text. Furthermore, add Pb to the trace element patterns.

Version 1:

Reviewer comments:

Reviewer #1

(Remarks to the Author)
The authors have satisfactorily addressed all of my comments and suggestions. The manuscript has been substantially improved through a more comprehensive contextualisation within the framework of recent literature on the deep carbon cycle. This study provides a timely and significant contribution to our understanding of deep carbon cycling and will be of broad interest to the scientific community. I therefore consider the manuscript suitable for publication in Nature Communications.

Sincerely,
Fabio Arzilli

Reviewer #2

(Remarks to the Author)
Thanks to the authors for carefully addressing my previous comments. I am happy to recommend the manuscript for publication.

Minor comment:
I suggest that the authors revise the framing statement in the abstract and introduction to better reflect the current debate on deep carbon recycling.
In the abstract and introduction, the authors state that existing models predict nearly complete decarbonation of subducted slabs before they reach sub-arc depths, particularly in hot subduction zones, thereby limiting the deep recycling of surface carbon. However, this statement appears to be too generalized, as several studies have suggested that significant amounts of carbon may survive devolatilization and be transported to deeper mantle depths. Therefore, this issue remains an active debate rather than a consensus view. The authors should revise this statement to better acknowledge the ongoing debate and avoid implying that current models uniformly support limited deep carbon recycling. A more balanced description would strengthen the introduction and provide a more accurate context for the significance of this study.

Open Access This Peer Review File is licensed under a Creative Commons Attribution 4.0 International License, which permits use, sharing, adaptation, distribution and reproduction in any medium or format, as long as you give appropriate credit to the original author(s) and the source, provide a link to the Creative Commons license, and indicate if changes were made.
In cases where reviewers are anonymous, credit should be given to 'Anonymous Referee' and the source.
The images or other third party material in this Peer Review File are included in the article's Creative Commons license, unless indicated otherwise in a credit line to the material. If material is not included in the article's Creative Commons license and your intended use is not permitted by statutory regulation or exceeds the permitted use, you will need to obtain permission directly from the copyright holder.
To view a copy of this license, visit https://creativecommons.org/licenses/by/4.0/

Revised Heavy Boron Isotopes Reveal Deep Carbon Recycling in Hot Subduction Zones

Corresponding Author: Dr Hai-Quan Liu

This file contains the comments from the handling editor and all reviewers, together with the authors' detailed responses to each comment.

REVIEWER COMMENTS

Reviewer #1 (Remarks to the Author):

Summary and main findings of the study: This study challenges existing models that predict near-complete decarbonation of subducted slabs before reaching sub-arc depths, especially in hot subduction zones. Using boron isotopes and geochemical data from olivine- hosted melt inclusions in El Barú adakites (Panama), the authors identify signatures of subducted carbonate-derived carbon rather than fluids from serpentinite.
Elevated δ11B values and high Ce/Pb ratios indicate that carbonate-rich lithologies can survive shallow decarbonation. Combined with regional CO2 flux estimates, the results suggest that over 60% of slab carbonate is retained and transported to greater depths, likely due to forearc dehydration limiting carbon loss.
Overall, the study demonstrates that significant carbonate recycling occurs even in hot subduction zones, supporting long-term deep carbon cycling since at least the late Archean.

Strengths and limitations of the study: This study addresses an interesting topic and provides a timely and relevant contribution to our understanding of deep carbon cycling. However, the results would benefit from a more comprehensive contextualization within the framework of recent literature. In particular, key studies such as Aiuppa et al. (2019), Werner et al. (2019), Farsang et al. (2021), and Arzilli et al. (2023) should be more thoroughly integrated. The findings of Arzilli et al. (2023) on metamorphic decarbonation and Farsang et al. (2021) on carbonate dissolution are especially pertinent and should be introduced earlier in the manuscript and discussed consistently throughout.
Moreover, the results from Arzilli et al. (2023) on metamorphic decarbonation and from Farsang et al. (2021) on carbonate dissolution should be presented in the Introduction section and discussed in throughout the manuscript. Some of the results presented here appear to confirm or build upon outcomes already reported by Arzilli et al. (2023). For this reason, the authors should more clearly articulate the rationale of the study and better highlight its novel contributions.
The overall structure of the manuscript also requires improvement. At present, the

distinction between the Introduction and the Results and Discussion sections is not sufficiently clear, which affects the readability of the paper. In addition, a dedicated Conclusions section is currently missing and should be included to effectively summarize the main findings and their broad implications.

Recommendation: I have provided several comments and suggestions throughout the manuscript that I hope will help strengthen the study. In my opinion, the manuscript has the potential to be suitable for publication in Nature Communications, but only after substantial revision.

Yours sincerely,
Fabio Arzilli

Detailed Comments

- Introduction

Lines 36-39: The authors stated: "*Flux-based estimates argue that most slab carbon is released at shallow depths—particularly in hot subduction zones (>330–360 °C GPa-1), where models predict near-complete decarbonation before the slab reaches sub-arc conditions*." Recent relevant articles are not considered here (Farsang et al., 2021; Arzilli et al., 2023). Particularly, recent models present a global arc-by-arc lithology-specific analysis of the magnitude of slab CO2 released purely by metamorphic decarbonation of carbonate- bearing sediment and basalt during subduction of altered oceanic crust (Arzilli et al., 2023). The authors have found that metamorphic decarbonation is highly efficient in low carbonate sediments, such as carbonated clay, and in carbonate-bearing basalts of altered oceanic crust, causing all of their CO2 to be removed (Arzilli et al., 2023). Sediments with medium and higher carbonate contents, such as chalk and limestone, are only partially decarbonated. The warmest slabs, such as Mexico and Cascadia, produce complete metamorphic decarbonation of carbonate-poor sediments beneath fore-arcs. Under more common intermediate-warm thermal regimes, and sediments with higher carbonate contents, such as most of the East Pacific subduction zones with carbonate-rich sediments (Columbia-Ecuador, Nicaragua, Guatemala-El Salvador, Costa Rica and Peru) only partial metamorphic decarbonation of carbonate minerals occurs at depths between ~80 and 170 km (~2.3 to 5.5 GPa) (see Figure attached).

- Fate of subducting carbonates at hot subduction systems

Lines 161-195: The discussion in this section does not adequately consider key studies that have quantified global volcanic CO2 emissions (e.g., Aiuppa et al., 2019; Werner et al., 2019). Incorporating these works would provide an essential framework for interpreting the results. In addition, the manuscript does not sufficiently address the fate of subducting carbonates in hot subduction zone settings. This aspect should be better contextualized by comparing CO2 outputs from volcanic arcs (Aiuppa et al., 2019; Werner et al., 2019) with estimates of CO2 input from sub-arc slabs (Farsang et al., 2021; Arzilli et al., 2023). Such a comparison is critical to properly constrain the role

of subduction processes in the deep carbon cycle and to place the study's findings into a broader geodynamic perspective.

References

Aiuppa, A., Fischer, T.P., Plank, T., Bani, P., 2019. CO2 flux emissions from the Earth's most actively degassing volcanoes, 2005–2015. Sci. Rep. 9, 1–17. https://doi.org/ 10.1038/s41598-019-41901-y.

Arzilli, F., Burton, M., La Spina, G., Macpherson, C.G., van Keken, P.E. and McCann, J., 2023. Decarbonation of subducting carbonate-bearing sediments and basalts of altered oceanic crust: Insights into recycling of CO2 through volcanic arcs. Earth and Planetary Science Letters, 602, p.117945.

Farsang, S., Louvel, M., Zhao, C., Mezouar, M., Rosa, A.D., Widmer, R.N., Feng, X., Liu, J. and Redfern, S.A., 2021. Deep carbon cycle constrained by carbonate solubility. Nature Communications, 12(1), p.4311.

Werner, C., Fischer, T.P., Aiuppa, A., Edmonds, M., Cardellini, C., Carn, S., Chiodini, G., Cottrell, E., Burton, M., Shinohara, H., Allard, P., 2019. Carbon dioxide emissions from subaerial volcanic regions: two decades in review. In: Orcutt, B.N., Daniel, I., Dasgupta, R. (Eds.), Deep Carbon Past to Present. Cambridge University Press, pp. 188–236.

Reviewer #2 (Remarks to the Author):

This study presents novel boron isotope and geochemical data from olivine-hosted melt inclusions in adakites from western Panama, providing strong evidence for efficient deep recycling of carbonates in a hot subduction zone. The manuscript is well structured, the dataset is robust, and the interpretations are generally convincing. I consider the manuscript suitable for publication after addressing the following minor comments.

I suggest moving the major and trace element figures from the Supplementary Information into the main text to improve data accessibility. In addition, Pb should be included in the trace element patterns.

It would also be valuable to further examine additional carbonatitic proxies, such as SiO_2, CaO, La/Yb, Zr/Hf, and Ti/Eu, to better constrain the potential carbonatite signature in the melt inclusion compositions. Including an additional figure would further enhance clarity and readability.

If possible, please include representative petrographic images of the melt inclusions in the supplementary information. Furthermore, presenting the major and trace element compositions of the host olivine would help to better constrain the stage of magma differentiation during which the melt inclusions were trapped.

Minor comments:
Lines 39–41: please note that carbonates in intraplate magma can also be derived from the deep mantle rather than subduction.
Lines 47–48: please check the sentence: “rendering the sub-arc mantle as the final major locus of slab carbon loss following fore-arc devolatilization.”
Figure 1b: this figure gives the false impression that all carbon can be subducted into the deep mantle. It is suggested to add more solidi of carbon-bearing materials in subduction zones.
Lines 72–75: it would be better to briefly describe how boron isotopes of olivine-hosted melt inclusions were measured.
Figure 2: please check the carbonatitic proxies: La/Yb, Zr/Hf, Ti/Eu.
Lines 364–368: please add a figure in the supplementary information comparing the measured B isotopes of the reference materials with their recommended values.
Supplementary information: I suggest moving the major and trace element figures from the supplementary information into the main text. Furthermore, add Pb to the trace element patterns.

Point-by-point response to reviewer #1

1. **Summary and main findings of the study:** This study challenges existing models that predict near-complete decarbonation of subducted slabs before reaching sub-arc depths, especially in hot subduction zones. Using boron isotopes and geochemical data from olivine- hosted melt inclusions in El Barú adakites (Panama), the authors identify signatures of subducted carbonate-derived carbon rather than fluids from serpentinite.

   Elevated $\delta^{11}B$ values and high Ce/Pb ratios indicate that carbonate-rich lithologies can survive shallow decarbonation. Combined with regional CO_2 flux estimates, the results suggest that over 60% of slab carbonate is retained and transported to greater depths, likely due to forearc dehydration limiting carbon loss.

   Overall, the study demonstrates that significant carbonate recycling occurs even in hot subduction zones, supporting long-term deep carbon cycling since at least the late Archean.

   We sincerely thank the reviewer for the constructive comments. The manuscript has been thoroughly revised and strengthened throughout in accordance with the reviewer's suggestions. Specifically, the Introduction (From this point onward, all text highlighted in red—including section headings, line numbers, and figure/table citations—corresponds to the relevant revisions in the track-changes version of the manuscript, unless otherwise specified) has been expanded to provide additional background information and to incorporate citations to relevant key studies. We have reorganized the Results and Discussion sections in accordance with the requirements of *Nature Communications*.

2. **Strengths and limitations of the study:** This study addresses an interesting topic and provides a timely and relevant contribution to our understanding of deep carbon cycling. However, the results would benefit from a more comprehensive contextualization within the framework of recent literature. In particular, key studies such as Aiuppa et al. (2019), Werner et al. (2019), Farsang et al. (2021), and Arzilli et al. (2023) should be more thoroughly integrated. The findings of Arzilli et al. (2023) on metamorphic decarbonation and Farsang et al. (2021) on carbonate dissolution are especially pertinent and should be introduced earlier in the manuscript and discussed consistently throughout. Moreover, the results from Arzilli et al. (2023) on metamorphic decarbonation and from Farsang et al. (2021) on carbonate dissolution should be presented in the Introduction section and discussed in throughout the manuscript. Some of the results presented here appear to confirm or build upon outcomes already reported by Arzilli et al. (2023). For this reason, the authors should more clearly articulate the rationale of the study and better highlight its novel contributions.

   Yes, this study was developed based on previous work proposing that hot subduction systems experience extensive devolatilization in the forearc region. We sincerely thank the reviewer for drawing our attention to these important studies, which have

enabled the revised manuscript to more comprehensively acknowledge the pioneering contributions made in this field. Building upon these previous datasets and interpretations, the revised manuscript further discusses and better constrains the mechanisms and processes governing carbon cycling in hot subduction systems. The Introduction has been strengthened by expanding the background discussion of subduction-zone carbon cycling based on Arzilli et al. (2023) and Farsang et al. (2021). In particular, we incorporated published carbon flux estimates derived from theoretical modelling, which indicate that, in hot subduction systems, most carbon loss may already occur in the forearc region (Lines 48 - 51).

In the Discussion section, building on the inference of high shallow carbon cycling efficiency in hot subduction systems proposed by Arzilli et al. (2023) and Farsang et al. (2021), we explore the processes responsible for the unusually low carbon cycling efficiency observed beneath western Panama. Specifically, we argue that the low efficiency results primarily from carbon escaping subduction-related decarbonation processes rather than being stored within the sub-arc lithospheric mantle (Lines 258 - 266). We further propose that this low carbon cycling efficiency reflects intense early-stage slab devolatilization, which suppresses fluid-assisted metamorphic reactions as well as dissolution- and melting-driven decarbonation processes in both the forearc and at greater sub-arc depths (Lines 267 - 297). Finally, based on the geochemical similarities between magmas from modern and Archean hot subduction systems and the MIs from western Panama, we extend this interpretation of deep carbon recycling to these analogous subduction settings (Lines 298 - 316).

Following the reviewer's recommendation of Aiuppa et al. (2019) and Werner et al. (2019) on global volcanic CO_2 emissions, we identified carbon emission estimates for forearc and arc-front volcanoes in Cascadia from Werner et al. (2019) and Fischer et al. (2019), calculated the carbon flux of the Cascadia subduction system, and compared it directly with that of western Panama (Lines 303 - 310). For the forearc and arc-front carbon emissions of the southern Central American Volcanic Arc (South CAVA), we retained the estimates of Barry et al. (2019) (Lines 256 - 258), as this study was published in the same year as Aiuppa et al. (2019) and Werner et al. (2019) and therefore provides comparable temporal relevance.

3. The overall structure of the manuscript also requires improvement. At present, the distinction between the Introduction and the Results and Discussion sections is not sufficiently clear, which affects the readability of the paper. In addition, a dedicated Conclusions section is currently missing and should be included to effectively summarize the main findings and their broad implications.

The Introduction has been expanded to provide additional background information. According to the structural requirements of *Nature Communications*, a new Results section has been added to present the carbonate-related geochemical characteristics documented in this study (Lines 121 - 137), as well as the genetic interpretation of

the light (Lines 179 - 197) and heavy boron isotope signatures (Lines 199 - 247). The original section titled "Fate of subducting carbonates at hot subduction systems" has been revised and retitled as "Discussion" to better emphasize the broader implications of this work, particularly its relevance to deep carbon subduction in both modern and Archean hot subduction systems, and the implications for carbonate signatures in modern ocean island basalts (Lines 298 - 316). The Discussion has been further strengthened to more fully elaborate on the fate of subducted carbonates.

In addition, a Conclusions section (shown in green text below) was added to summarize the key findings and highlight the main implications of this study. However, we note that the standard *Nature Communications* template does not include a separate Conclusions section, and instead recommends placing concluding statements at the end of the Introduction (Lines 103 - 110).

Conclusions

This study presents melt inclusion data from arc front El Barú adakites in the southern Central American Volcanic Arc and from forearc Shido-Shima high-Mg andesites in the Setouchi Volcanic Belt, and integrates these datasets with geochemical data from modern and Archean hot subduction systems to derive the following conclusions:

1) Low $\delta^{11}B$ values (-13.4±2.1‰ to -2.9±3.8‰) associated with low trace element ratios for melt inclusions of modern hot subduction systems indicate melting of subducted oceanic crust with a contribution from depleted mantle. In contrast, moderate $\delta^{11}B$ values (-0.7 ± 3.6‰ to +7.8 ± 4.8‰) coupled with intermediate trace element ratios are interpreted to reflect a contribution from serpentinite-derived fluids, whereas moderate to high $\delta^{11}B$ values (+0.3 ± 3.4‰ to +23.2 ± 2.7‰) accompanied by high trace element ratios are best explained by a contribution from carbonatitic melts.

2) The low carbon cycling efficiency inferred for western Panama (11–38%) and Cascadia (~24%), together with their adakitic geochemical signatures indicative of slab melting, suggests that extensive forearc fluid loss in hot subduction systems suppresses efficient decarbonation through metamorphic reactions, dissolution, and melting beneath the deeper forearc and the arc.

3) The geochemical similarity between Archean heavy-B tonalite-trondhjemite-granodiorite suites and modern adakitic melt inclusions implies that deep carbon recycling associated with subduction processes had already been established by the late Archean.

4. Recommendation: I have provided several comments and suggestions throughout the manuscript that I hope will help strengthen the study. In my opinion, the manuscript has the potential to be suitable for publication in Nature Communications, but only after substantial revision.

We sincerely thank the reviewer for the thorough evaluation of the manuscript and

for the constructive and insightful comments. In response to these suggestions, we have substantially strengthened the relevant sections of the manuscript. Detailed point-by-point responses are provided below.

Detailed Comments

5. Introduction

Lines 36-39: The authors stated: "Flux-based estimates argue that most slab carbon is released at shallow depths—particularly in hot subduction zones (>330–360 °C GPa-1), where models predict near-complete decarbonation before the slab reaches sub-arc conditions." Recent relevant articles are not considered here (Farsang et al., 2021; Arzilli et al., 2023). Particularly, recent models present a global arc-by-arc lithology-specific analysis of the magnitude of slab CO_2 released purely by metamorphic decarbonation of carbonate- bearing sediment and basalt during subduction of altered oceanic crust (Arzilli et al., 2023). The authors have found that metamorphic decarbonation is highly efficient in low carbonate sediments, such as carbonated clay, and in carbonate-bearing basalts of altered oceanic crust, causing all of their CO_2 to be removed (Arzilli et al., 2023). Sediments with medium and higher carbonate contents, such as chalk and limestone, are only partially decarbonated. The warmest slabs, such as Mexico and Cascadia, produce complete metamorphic decarbonation of carbonate-poor sediments beneath fore-arcs. Under more common intermediate-warm thermal regimes, and sediments with higher carbonate contents, such as most of the East Pacific subduction zones with carbonate-rich sediments (Columbia-Ecuador, Nicaragua, Guatemala-El Salvador, Costa Rica and Peru) only partial metamorphic decarbonation of carbonate minerals occurs at depths between ~80 and 170 km (~2.3 to 5.5 GPa) (see Figure attached).

We fully agree with the reviewer and acknowledge the importance of the recommended studies on global subduction-zone carbon fluxes, particularly those constraining carbon budgets in cold to moderately warm subduction systems. However, these previous studies did not include carbon flux estimate for the western Panama subduction system.

Based on the occurrence of forearc adakites and high-Mg andesites in western Panama and Setouchi, respectively—both widely interpreted as products of slab melting—the Setouchi and western Panama subduction systems can be inferred to represent relatively hot subduction settings, potentially even hotter than Cascadia and Mexico (Fig. 1 and Supplementary Fig. 3). Such exceptionally high slab thermal gradients may result from: 1) the immediate subduction (initiated at ~17 Ma) of Shikoku Basin (30–15 Ma) beneath Setouchi, which has been linked to the generation of forearc high-Mg andesites (Tatsumi, 2006); and 2) thermal modification of the subducting oceanic slab beneath western Panama associated with the influence of the Galápagos mantle plume (Gazel et al., 2011; Messling et al., 2023). Under such elevated thermal conditions, serpentinized mantle is expected to undergo rapid and extensive dehydration at relatively shallow forearc depths

(Leeman et al., 1994; Shaw et al., 2003). This early fluid loss may suppress fluid-assisted metamorphic reactions, carbonate dissolution, and slab-melting-related decarbonation processes in both the forearc and sub-arc regions (Lines 280 - 292). As a consequence, carbon may bypass shallow decarbonation and be transported to greater depths.

Alternatively, carbonates may react with serpentinite or fluids within the slab and transform into Mg-rich carbonate phases (e.g., magnesite), which possess a wider pressure–temperature stability field and substantially lower solubility than Ca-rich carbonates (carbonate solubility decreases by at least two orders of magnitude with increasing Mg content; Farsang et al., 2021). Such a mechanism could also facilitate carbonate survival through shallow subduction environments. However, we chose not to expand this aspect in the revised manuscript because our interpretation emphasizes fluid limitation as the primary control on suppressing decarbonation, and maintaining focus on this mechanism allows the discussion to remain concise and coherent.

Regarding sediment diapirism-related decarbonation, we did not consider this mechanism in the revised manuscript because the inferred slab-top thermal gradients already reach the pressure–temperature conditions required for melting, making this process less relevant in the context of our study.

6. Fate of subducting carbonates at hot subduction systems

Lines 161-195: The discussion in this section does not adequately consider key studies that have quantified global volcanic CO_2 emissions (e.g., Aiuppa et al., 2019; Werner et al., 2019). Incorporating these works would provide an essential framework for interpreting the results. In addition, the manuscript does not sufficiently address the fate of subducting carbonates in hot subduction zone settings. This aspect should be better contextualized by comparing CO_2 outputs from volcanic arcs (Aiuppa et al., 2019; Werner et al., 2019) with estimates of CO_2 input from sub-arc slabs (Farsang et al., 2021; Arzilli et al., 2023). Such a comparison is critical to properly constrain the role of subduction processes in the deep carbon cycle and to place the study's findings into a broader geodynamic perspective.

We thank the reviewer for the constructive comments. Following the studies of Werner et al. (2019) and Fischer et al. (2019), we have incorporated estimates of carbon cycling efficiency for the Cascadia subduction system and included a comparative discussion with that of western Panama (Lines 303 - 306).

The primary objective of this study is to evaluate whether slabs in hot subduction systems can bypass forearc decarbonation, based on the geochemical signatures preserved in MIs from the western Panama arc front. Combined with carbon flux estimates, the carbonate-related geochemical characteristics recorded by arc front adakitic MIs provide evidence that, in hot subduction settings, a substantial proportion of subducted carbonate can bypass forearc decarbonation and be

transported to greater depths. We regard this as one of the key findings and novel contributions of the present study. Considering the geochemical similarities and comparable carbon flux estimates between western Panama and other hot subduction systems, the manuscript further extends the interpretation derived from western Panama to both modern and Archean hot subduction regimes, which forms the broader implication of this study (Lines 298 - 316). At the same time, the manuscript also presents geochemical data from relatively colder subduction systems (e.g., Izu–Bonin and South Sandwich), which display signatures characteristic of serpentinite-fluid influence (Figure 5) rather than carbonate melt contributions. Therefore, our interpretation is not intended to be universally applicable to these colder subduction environments.

Previous studies in the south CAVA have extensively investigated the imbalance between slab carbon input and volcanic carbon output based on carbon emissions from forearc springs and arc volcanoes (Figure below) (Barry et al., 2019; Freundt et al., 2014; Jarrard, 2003; Shaw et al., 2003). Accordingly, we cited these published estimates in the manuscript and did not repeat this line of discussion. In particular, for the carbon flux estimate in western Panama, we adopted the calculations reported by Barry et al. (2019), which themselves are based on the carbon input constraints of Shaw et al. (2003). Given that Barry et al. (2019) was published in the same year as Aiuppa et al. (2019) and Werner et al. (2019), we consider the dataset to have comparable temporal relevance, although the latter studies also reported broadly similar volcanic carbon emission estimates for western Panama.

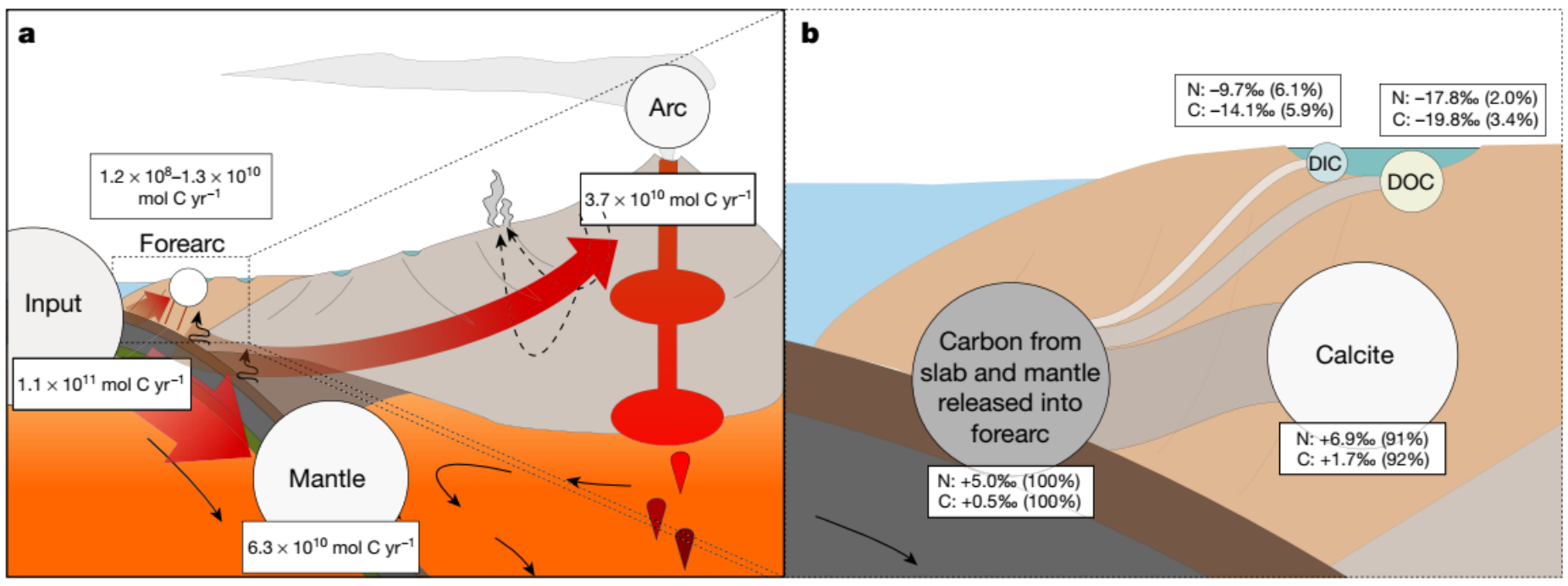

Figure 4 from Barry et al. (2019), illustrating the imbalance between subducted carbon input and carbon output from forearc springs and arc volcanism in the southern Central American Volcanic Arc.

7. References

Aiuppa, A., Fischer, T.P., Plank, T., Bani, P., 2019. CO2 flux emissions from the Earth's most actively degassing volcanoes, 2005–2015. Sci. Rep. 9, 1–17. https://doi.org/ 10.1038/s41598-019-41901-y.

Arzilli, F., Burton, M., La Spina, G., Macpherson, C.G., van Keken, P.E. and McCann, J., 2023. Decarbonation of subducting carbonate-bearing sediments and basalts of altered oceanic crust: Insights into recycling of CO2 through volcanic arcs. Earth and Planetary Science Letters, 602, p.117945.

Farsang, S., Louvel, M., Zhao, C., Mezouar, M., Rosa, A.D., Widmer, R.N., Feng, X., Liu, J. and Redfern, S.A., 2021. Deep carbon cycle constrained by carbonate solubility. Nature Communications, 12(1), p.4311.
Werner, C., Fischer, T.P., Aiuppa, A., Edmonds, M., Cardellini, C., Carn, S., Chiodini, G., Cottrell, E., Burton, M., Shinohara, H., Allard, P., 2019. Carbon dioxide emissions from subaerial volcanic regions: two decades in review. In: Orcutt, B.N., Daniel, I., Dasgupta, R. (Eds.), Deep Carbon Past to Present. Cambridge University Press, pp. 188–236.

We thank the reviewer for recommending these references. Building on these four studies, we identified additional relevant literature and incorporated the corresponding datasets into the revised manuscript to further expand the discussion of carbon cycling in hot subduction systems (Please see responses #2, 5, and 6 for more information).

Point-by-Point response to reviewer #2

1. This study presents novel boron isotope and geochemical data from olivine-hosted melt inclusions in adakites from western Panama, providing strong evidence for efficient deep recycling of carbonates in a hot subduction zone. The manuscript is well structured, the dataset is robust, and the interpretations are generally convincing. I consider the manuscript suitable for publication after addressing the following minor comments.

   We thank the reviewer for the helpful comments. Following the suggestions from Reviewer #1, we have reorganized the structure of the manuscript to better highlight and communicate our key findings.

2. I suggest moving the major and trace element figures from the Supplementary Information into the main text to improve data accessibility. In addition, Pb should be included in the trace element patterns.

   The major and trace element diagrams have been moved into the main text (Figs. 2 and 3) to improve data accessibility. In the original primitive mantle–normalized trace element diagrams, Pb data for the MIs were not presented because Pb data are generally unavailable for Archean TTGs used for comparison. In the revised manuscript, Pb data for the MIs are now included. Regarding the negative Pb anomaly observed in the multi-trace element diagrams, one possible explanation is sequestration of Pb into sulfides. However, if sulfide fractionation were responsible for the Pb depletion, Ni concentrations in the corresponding MIs should also decrease significantly, given the strong compatibility of Ni in sulfide phases. In contrast, Ni concentrations in the MIs (159–381 μg/g) (Supplementary Data 1; From this point onward, all text highlighted in red—including section headings, line numbers, and figure/table citations—corresponds to the relevant revisions in the track-changes version of the manuscript, unless otherwise specified) are comparable to those of the host adakites (114–197 μg/g) (Liu et al., 2024), arguing against substantial sulfide segregation. Moreover, other carbonatitic proxies likewise display elevated values, further supporting a carbonate contribution (Figure 5b-d). We therefore suggest that the negative Pb anomaly is more plausibly interpreted as reflecting compositional control exerted by carbonatitic melts in the mantle source.

3. It would also be valuable to further examine additional carbonatitic proxies, such as SiO2, CaO, La/Yb, Zr/Hf, and Ti/Eu, to better constrain the potential carbonatite signature in the melt inclusion compositions. Including an additional figure would further enhance clarity and readability.

   We thank the reviewer for the constructive suggestion. We have evaluated $\delta^{11}B$ against SiO_2, CaO, CaO/Al_2O_3, Zr/Hf (Figures below), La/Yb, Eu/Ti, and Th/Zr (Fig. 5). The results show that $\delta^{11}B$ does not display the expected correlations with SiO_2, CaO, CaO/Al_2O_3, or Zr/Hf. By contrast, $\delta^{11}B$ generally increases with increasing

La/Yb, Eu/Ti, and Th/Zr, consistent with the expected behavior of carbonate involvement. The absence of a clear $\delta^{11}B$–SiO_2 relationship may reflect uncertainties introduced during multi-parameter correction using Petrolog3, where the corrected major element compositions can deviate from their original values due to non-unique parameter selection (Danyushevsky and Plechov, 2011). In addition, silica-rich carbonatitic melts reacting with peridotite during ascent may form clinopyroxene that preferentially incorporates Ca, consistent with the overall low CaO/Al_2O_3 of the MIs (Supplementary Data 1). This process may buffer or obscure any positive correlation between $\delta^{11}B$ and CaO or CaO/Al_2O_3 that would otherwise be expected from the addition of Ca-rich, heavy-B carbonatitic melts.
Although heavy-B carbonatitic melts could potentially induce Zr–Hf fractionation, both Zr and Hf are more compatible in silicate melts than in carbonatitic melts (Veksler et al., 1998) and may be further overprinted by melt–peridotite reactions that stabilize clinopyroxene, which can modify Zr/Hf systematics (Münker et al., 2004). This may explain the lack of a clear positive correlation between $\delta^{11}B$ and Zr/Hf. Accordingly, in the revised manuscript we have added cross-plots of $\delta^{11}B$ versus La/Yb, Eu/Ti, and Th/Zr (Fig. 5) to better evaluate potential source-related controls.

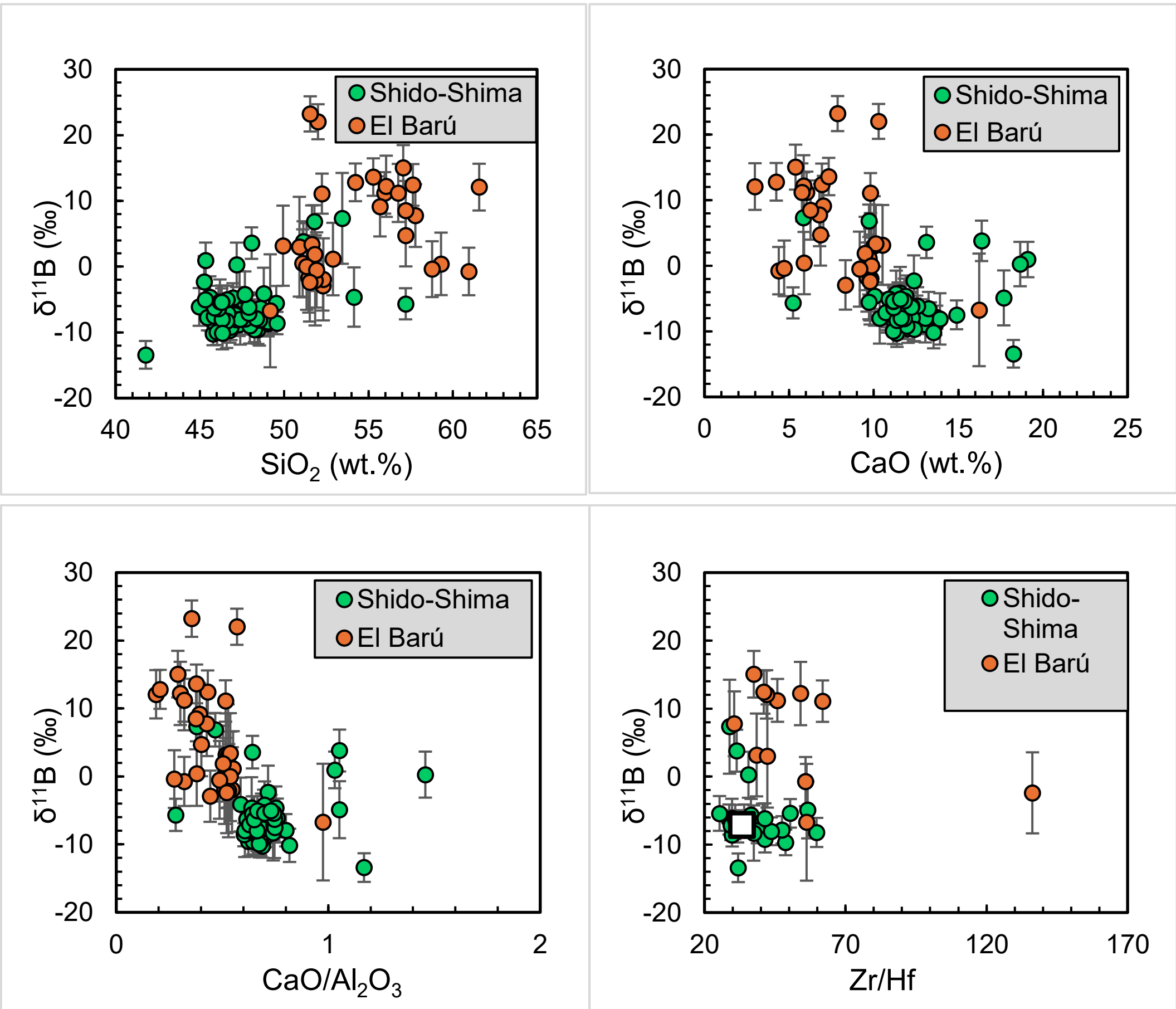

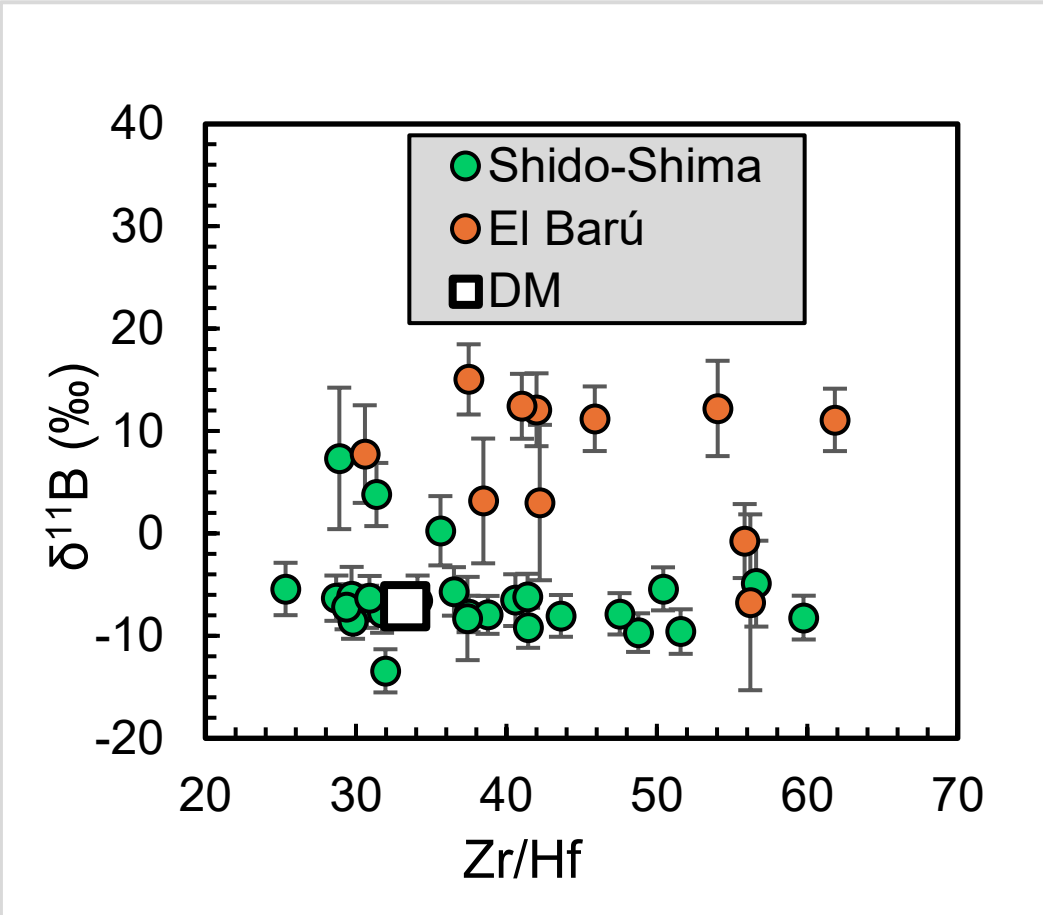

4. If possible, please include representative petrographic images of the melt inclusions in the supplementary information. Furthermore, presenting the major and trace element compositions of the host olivine would help to better constrain the stage of magma differentiation during which the melt inclusions were trapped.

Representative photomicrographs of MIs and their host olivine crystals have been provided in the Supplementary Information (Supplementary Figure 4). Given that the inclusion of additional MI data from Setouchi has substantially increased the overall dataset, we prefer not to expand the dataset further.
Based on Fo–Ni systematics (Figure below), our olivine data indicate a chemical signature consistent with equilibrium between slab-derived melts and pyroxenites, which represents a distinct petrogenetic process and therefore falls outside the main focus of this study. In addition, the olivine Fo contents are generally high (>85; Figure below and Supplementary Data 1), suggesting crystallization during relatively early stages of magma evolution. As a result, the hosted MIs likely record relatively primitive melt compositions.

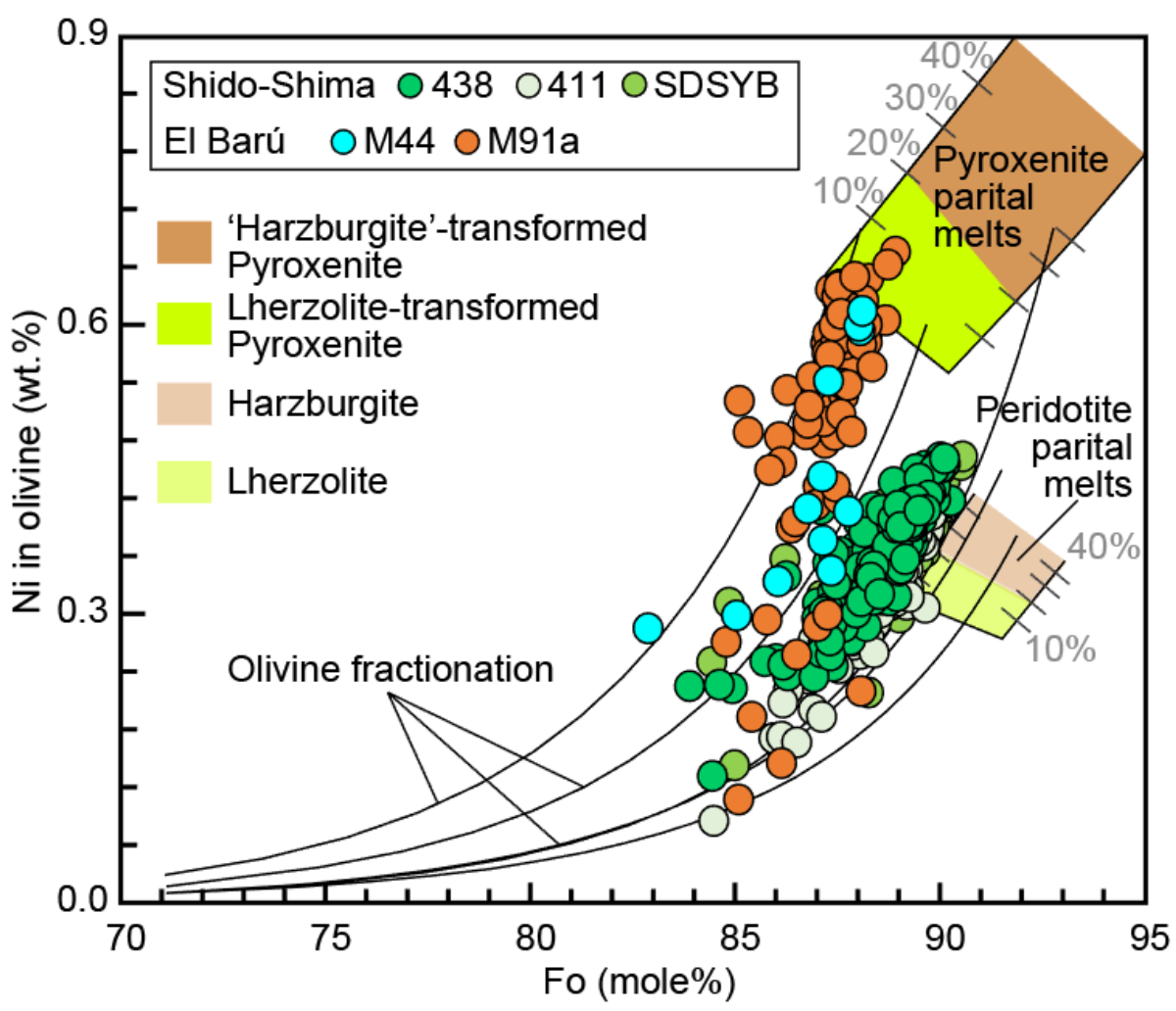

Plot of olivine Ni versus Fo (=Mg/(Mg+Fe)). High Fo and Ni contents of olivine hosting the MIs indicate equilibration between slab-derived melts and pyroxenitic lithologies. Fields of

pyroxenite and peridotite melts are based on calculations of Straub et al. (2008).

Minor comments:

5. Lines 39–41: please note that carbonates in intraplate magma can also be derived from the deep mantle rather than subduction.
   Thanks. This sentence has been rephrased by adding words "derived from recycled mantle domains" (Line 52).

6. Lines 47–48: please check the sentence: "rendering the sub-arc mantle as the final major locus of slab carbon loss following fore-arc devolatilization."
   Thank you for this helpful reminder. Our previous wording was not sufficiently precise, as recent experimental studies have shown that slab melting at depths of 300–700 km can also result in significant decarbonation (Thomson et al., 2016). Accordingly, we have revised the sentence by replacing "the final" with "a major" (Line 61).

7. Figure 1b: this figure gives the false impression that all carbon can be subducted into the deep mantle. It is suggested to add more solidi of carbon-bearing materials in subduction zones.
   The solidi for decarbonation of subducted basalts and sediments from refs (Kerrick and Connolly, 2001a, b) have been added to Fig. 1.

8. Lines 72–75: it would be better to briefly describe how boron isotopes of olivine-hosted melt inclusions were measured.

   Because, in response to Reviewer 1's suggestion, we have reorganized the manuscript to present the analytical results first, a brief description of the B isotope analytical method here would be somewhat redundant. To maintain a more concise structure, we therefore prefer to keep the B isotope analytical procedures in the Methods section.

9. Figure 2: please check the carbonatitic proxies: La/Yb, Zr/Hf, Ti/Eu.
   Thank you. We have added cross-plots of $\delta^{11}B$ versus La/Yb, Eu/Ti, and Th/Zr (Figure 5; Lines 209 - 212 and 231 - 232). Please see Response #3 for further details.

10. Lines 364–368: please add a figure in the supplementary information comparing the measured B isotopes of the reference materials with their recommended values.

    A figure comparing the measured standards with the recommended values has been added to the Supplementary Information (Lines 398 - 402; Supplementary Figure 5).

11. Supplementary information: I suggest moving the major and trace element figures from the supplementary information into the main text. Furthermore, add Pb to the trace element patterns.

Done! (Figs 2 and 3) Please see response #2 to reviewer #2 for more details.

Barry, P.H., de Moor, J.M., Giovannelli, D., Schrenk, M., Hummer, D.R., Lopez, T., Pratt, C.A., Segura, Y.A., Battaglia, A., Beaudry, P., Bini, G., Cascante, M., d'Errico, G., di Carlo, M., Fattorini, D., Fullerton, K., Gazel, E., González, G., Halldórsson, S.A., Iacovino, K., Ilanko, T., Kulongoski, J.T., Manini, E., Martínez, M., Miller, H., Nakagawa, M., Ono, S., Patwardhan, S., Ramírez, C.J., Regoli, F., Smedile, F., Turner, S., Vetriani, C., Yücel, M., Ballentine, C.J., Fischer, T.P., Hilton, D.R., Lloyd, K.G., 2019. Forearc carbon sink reduces long-term volatile recycling into the mantle. Nature 568, 487-492.

Danyushevsky, L.V., Plechov, P., 2011. Petrolog3: Integrated software for modeling crystallization processes. Geochemistry, Geophysics, Geosystems 12.

Fischer, T.P., Arellano, S., Carn, S., Aiuppa, A., Galle, B., Allard, P., Lopez, T., Shinohara, H., Kelly, P., Werner, C., Cardellini, C., Chiodini, G., 2019. The emissions of CO2 and other volatiles from the world's subaerial volcanoes. Scientific Reports 9, 18716.

Freundt, A., Grevemeyer, I., Rabbel, W., Hansteen, T.H., Hensen, C., Wehrmann, H., Kutterolf, S., Halama, R., Frische, M., 2014. Volatile (H2O, CO2, Cl, S) budget of the Central American subduction zone. International Journal of Earth Sciences 103, 2101-2127.

Gazel, E., Hoernle, K., Carr, M.J., Herzberg, C., Saginor, I., den Bogaard, P.v., Hauff, F., Feigenson, M., Swisher, C., 2011. Plume–subduction interaction in southern Central America: Mantle upwelling and slab melting. Lithos 121, 117-134.

Jarrard, R.D., 2003. Subduction fluxes of water, carbon dioxide, chlorine, and potassium. Geochemistry, Geophysics, Geosystems 4.

Kerrick, D.M., Connolly, J.A.D., 2001a. Metamorphic devolatilization of subducted marine sediments and the transport of volatiles into the Earth's mantle. Nature 411, 293-296.

Kerrick, D.M., Connolly, J.A.D., 2001b. Metamorphic devolatilization of subducted oceanic metabasalts: implications for seismicity, arc magmatism and volatile recycling. Earth and Planetary Science Letters 189, 19-29.

Leeman, W.P., Carr, M.J., Morris, J.D., 1994. Boron geochemistry of the Central American Volcanic Arc: Constraints on the genesis of subduction-related magmas. Geochimica et Cosmochimica Acta 58, 149-168.

Liu, H.-Q., Tian, F., Hoernle, K., Li, J., Huang, X.-L., Zhang, L., Bindeman, I., Xu, Y.-G., 2024. Molybdenum isotope insights into recycling of wedge serpentinite and fore-arc crust in subduction zones. Chemical Geology 665, 122302.

Messling, N., Wörner, G., Willbold, M., 2023. Ancient mantle plume components constrained by tungsten isotope variability in arc lavas. Geochemical Perspectives Letters 26, 31-35.

Münker, C., Wörner, G., Yogodzinski, G., Churikova, T., 2004. Behaviour of high field strength elements in subduction zones: constraints from Kamchatka–Aleutian arc lavas. Earth and Planetary Science Letters 224, 275-293.

Shaw, A.M., Hilton, D.R., Fischer, T.P., Walker, J.A., Alvarado, G.E., 2003. Contrasting He–C relationships in Nicaragua and Costa Rica: insights into C cycling through subduction zones. Earth and Planetary Science Letters 214, 499-513.

Straub, S.M., LaGatta, A.B., Martin-Del Pozzo, A.L., Langmuir, C.H., 2008. Evidence from high-Ni olivines for a hybridized peridotite/pyroxenite source for orogenic andesites from the central Mexican Volcanic Belt. Geochemistry, Geophysics, Geosystems 9, Q03007.

Tatsumi, Y., 2006. High-Mg Andesites In The Setouchi Volcanic Belt, Southwestern Japan: Analogy

to Archean Magmatism and Continental Crust Formation? Annual Review of Earth & Planetary Science 34, 467-499.
Thomson, A.R., Walter, M.J., Kohn, S.C., Brooker, R.A., 2016. Slab melting as a barrier to deep carbon subduction. Nature 529, 76-79.
Veksler, I.V., Petibon, C., Jenner, G.A., Dorfman, A.M., Dingwell, D.B., 1998. Trace Element Partitioning in Immiscible Silicate–Carbonate Liquid Systems: an Initial Experimental Study Using a Centrifuge Autoclave. Journal of Petrology 39, 2095-2104.

Heavy Boron Isotopes Reveal Deep Carbon Recycling in Hot Subduction Zones

Corresponding Author: Dr Hai-Quan Liu

R2

This file contains the comments from all reviewers together with the authors' detailed responses to each comment.

REVIEWER COMMENTS

Reviewer #1 (Remarks to the Author):

The authors have satisfactorily addressed all of my comments and suggestions. The manuscript has been substantially improved through a more comprehensive contextualisation within the framework of recent literature on the deep carbon cycle. This study provides a timely and significant contribution to our understanding of deep carbon cycling and will be of broad interest to the scientific community. I therefore consider the manuscript suitable for publication in Nature Communications.

Sincerely,
Fabio Arzilli

Reviewer #2 (Remarks to the Author):

Thanks to the authors for carefully addressing my previous comments. I am happy to recommend the manuscript for publication.

Minor comment:
I suggest that the authors revise the framing statement in the abstract and introduction to better reflect the current debate on deep carbon recycling.
In the abstract and introduction, the authors state that existing models predict nearly complete decarbonation of subducted slabs before they reach sub-arc depths, particularly in hot subduction zones, thereby limiting the deep recycling of surface carbon. However, this statement appears to be too generalized, as several studies have suggested that significant amounts of carbon may survive devolatilization and be transported to deeper mantle depths. Therefore, this issue remains an active debate rather than a consensus view. The authors should revise this statement to better acknowledge the ongoing debate and avoid implying that current models uniformly support limited deep carbon recycling. A more balanced description would strengthen the introduction and provide a more accurate context for the significance of this study.

Point-by-point response to reviewer #1

1. The authors have satisfactorily addressed all of my comments and suggestions. The manuscript has been substantially improved through a more comprehensive contextualisation within the framework of recent literature on the deep carbon cycle. This study provides a timely and significant contribution to our understanding of deep carbon cycling and will be of broad interest to the scientific community. I therefore consider the manuscript suitable for publication in Nature Communications.

   We sincerely thank the reviewer for the positive assessment of our revised manuscript. We are particularly grateful for the constructive comments and suggestions provided during the first round of review, which helped us substantially improve the manuscript and place our findings within a broader context of deep carbon cycling. We greatly appreciate the reviewer's time and effort throughout the review process.

Point-by-Point response to reviewer #2

1. Minor comment:

   I suggest that the authors revise the framing statement in the abstract and introduction to better reflect the current debate on deep carbon recycling.

   In the abstract and introduction, the authors state that existing models predict nearly complete decarbonation of subducted slabs before they reach sub-arc depths, particularly in hot subduction zones, thereby limiting the deep recycling of surface carbon. However, this statement appears to be too generalized, as several studies have suggested that significant amounts of carbon may survive devolatilization and be transported to deeper mantle depths. Therefore, this issue remains an active debate rather than a consensus view. The authors should revise this statement to better acknowledge the ongoing debate and avoid implying that current models uniformly support limited deep carbon recycling. A more balanced description would strengthen the introduction and provide a more accurate context for the significance of this study.

   We thank the reviewer for this important suggestion. We agree that the fate of subducted carbon in hot subduction systems remains actively debated and that our previous framing did not sufficiently acknowledge the contrasting views in the literature. We have therefore revised both the Abstract and the first paragraph of the Introduction to provide a more balanced context for our study.

   In the revised Abstract, we now explicitly frame the issue as an ongoing debate: "The efficiency of deep carbon recycling in hot subduction systems remains debated, with contrasting models predicting either extensive shallow decarbonation or substantial carbonate retention within the subducted slab."

   In the revised Introduction, we have incorporated previous experimental and theoretical studies showing that substantial carbonate may remain stable and survive devolatilization even along relatively warm slab geotherms. We contrast these studies with models involving externally derived fluids and flux-based estimates that suggest extensive carbon release at shallow depths in hot subduction zones. In addition, we have incorporated independent evidence from intraplate continental basalts that preserves signatures of recycled Proterozoic to Archean carbonates in the mantle. These observations provide geological evidence for the long-term survival of deeply recycled carbonate and further highlight the apparent discrepancy between some theoretical predictions of extensive shallow decarbonation and observations from the geological record.

   Together, these revisions acknowledge the range of current views on carbon retention and decarbonation in hot subduction systems, rather than implying a consensus that deep carbon recycling is necessarily limited. We thank the reviewer again for this suggestion, which has helped us provide a more balanced framework for the significance of our study.
