# Supplementary material for: Heavy boron isotopes reveal deep carbon recycling in hot subduction zones
Source: Nat Commun. 2026 Sep 24;17:11104. doi: 10.1038/s41467-026-78096-6 (PMC13696696; doi:10.1038/s41467-026-78096-6)
Supplement: Supplementary file 2 [file 41467_2026_78096_MOESM1_ESM.pdf]

Supplementary Materials for

Heavy Boron Isotopes Reveal Deep Carbon Recycling in Hot Subduction Zones

Hai-Quan Liu[1, 2, 3]*, Feng Tian[1], Miao-Hong He[1], Ze-Xian Cui[1], Le Zhang[1], Peng-Li He[1], Lu-Bing Hong[4], Xiao-Long Huang[1, 2, 3], Ilya Bindeman[5], Kaj Hoernle[6, 7], Yi-Gang Xu[1, 2, 3]*

[1]State Key Laboratory of Deep Earth Processes and Resources, Guangzhou Institute of Geochemistry, Chinese Academy of Sciences, Guangzhou 510640, China

[2]Guangdong research center for strategic metals and green utilization

[3]Southern Marine Science and Engineering Guangdong Laboratory (Guangzhou), Guangzhou 511458, China

[4]School of Earth Sciences, Guilin University of Technology, Guilin 541006, China

[5]Department of Earth Sciences, University of Oregon, Eugene, OR 97403-1272, USA

[6]GEOMAR Helmholtz Centre for Ocean Research Kiel, Wischhofstraße 1-3, 24148 Kiel, Germany.

[7]Institute of Geosciences, Ludewig-Meyn-Straße 10, University of Kiel, 24118 Kiel, Germany

Corresponding authors: Hai-Quan Liu (liuhaiquan@gig.ac.cn); Yi-Gang Xu (yigangxu@gig.ac.cn)

Supplementary Figures

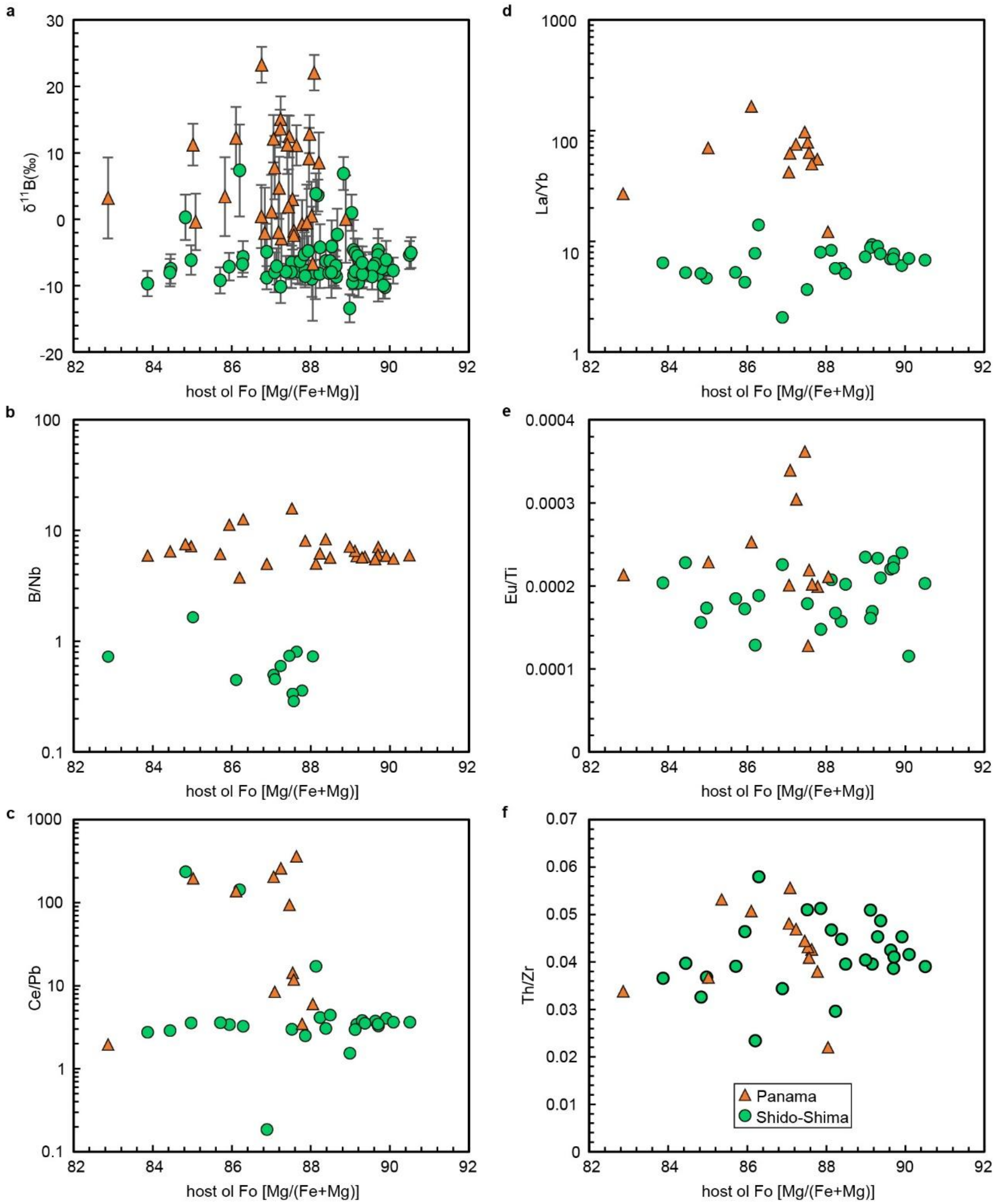

Supplementary Figure 1 | Plots between host olivine Fo contents and trace element ratios. a, b, c, d, e, and **f** The absence of a correlation between host olivine Fo contents (=Mg/(Fe+Mg)) and trace-element ratios suggests that intracrustal differentiation exerted little influence on these geochemical proxies. Error bars represent 2SE. Data supporting this figure are provided in Supplementary Data 1.

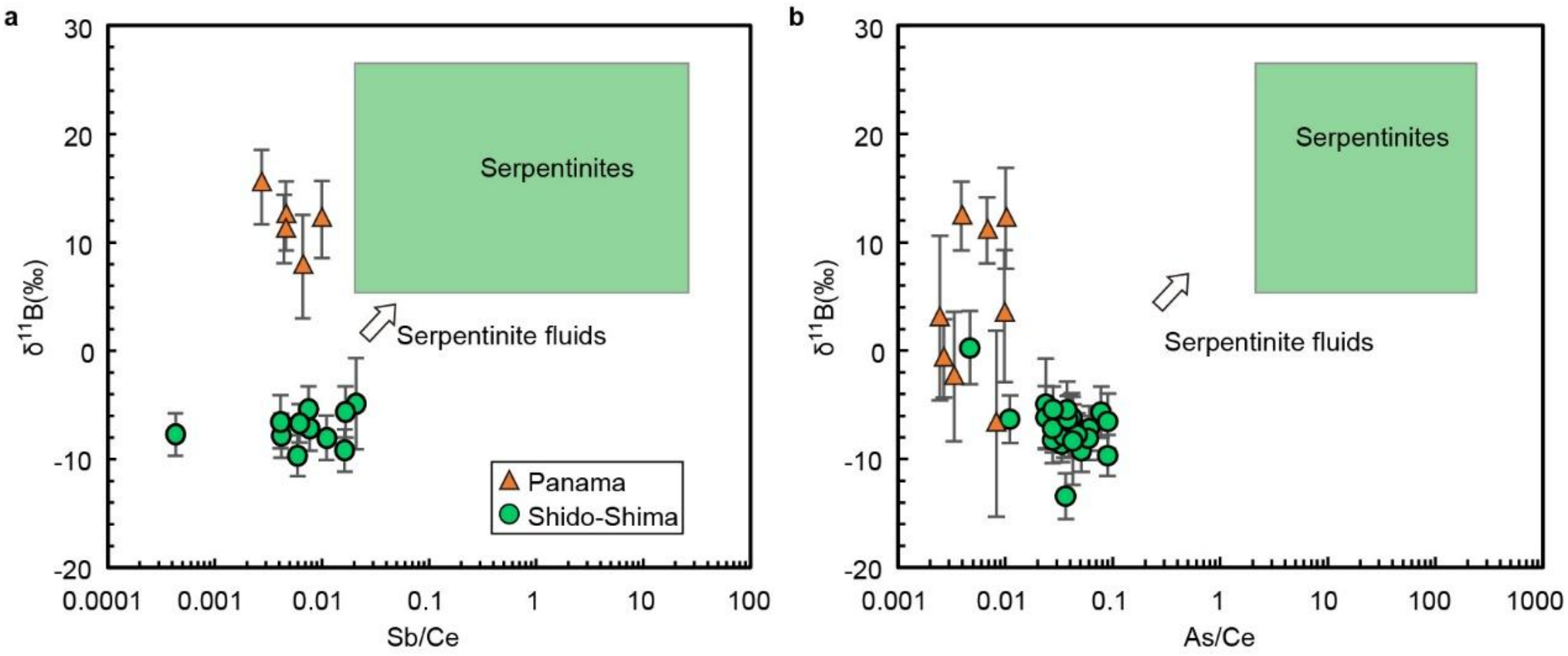

Supplementary Figure 2 | Plots of B isotope compositions versus serpentinite-sensitive trace element ratios. a and **b** The absence of concurrent enrichment in B isotopes and serpentinite-sensitive elements (Sb and As) argues against a dominant serpentinite contribution to the high $\delta^{11}B$ signatures of Panamanian and Shido-Shima melt inclusions. Serpentinite data are from refs [1,2]. Error bars represent 2SE. Data supporting this figure are provided in Supplementary Data 1.

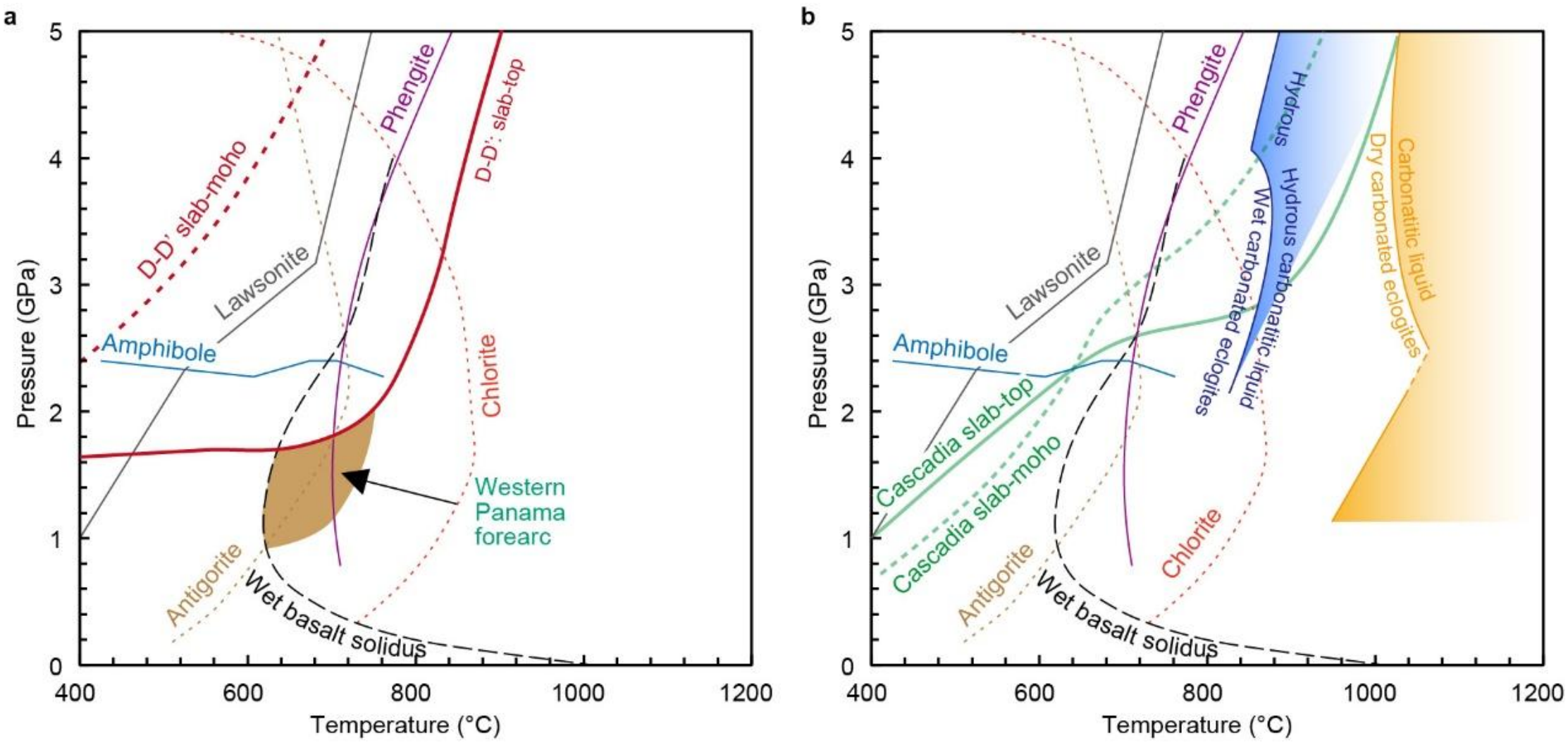

Supplementary Figure 3 | Comparison of slab thermal gradients and stability fields of hydrous minerals in the subducting slab. a The slab-top thermal gradients beneath western Panama (brown field) and southern Costa Rica (burgundy curve) [3] exceed the solidus of hydrous minerals such as lawsonite (grey line) , amphibole (blue line), and phengite (purple line) [4]. In southern Costa Rica, the slab–Moho thermal gradient [3] (dashed burgundy curve) intersects the solidi of antigorite (brown dotted line) and chlorite (orange dotted line) [4] at ~4.5 GPa. The wet basalt solidus is from ref [5]. **b** Both the Cascadia slab-Moho and slab-top thermal gradients [6] intersect the solidi of slab hydrous

minerals under arc conditions, and also intersect the wet carbonated eclogite solidus [7,8] at ~2.8 GPa and ~4 GPa, respectively. In contrast, under arc conditions, neither thermal gradient intersects the dry carbonated eclogite solidus [9].

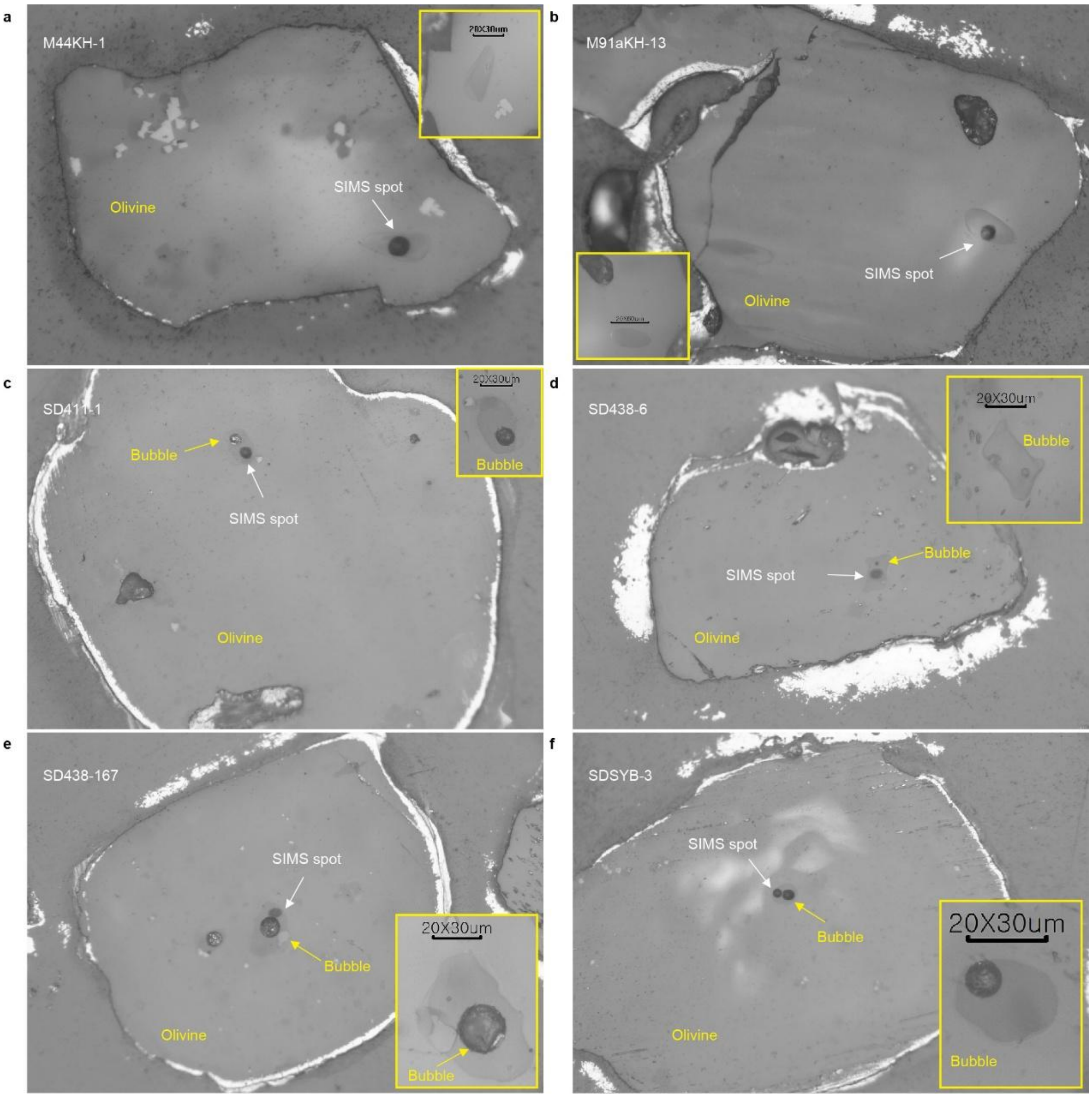

Supplementary Figure 4 | Representative photomicrographs of olivine-hosted melt inclusions from Panamanian and Shido-Shima lavas. a, b, c, d, e, and **f** Photomicrographs of melt inclusions from M44KH-1, M91aKH-13, SD411-1, SD438-6, SD438-167, and SDSYB-3 acquired after SIMS analysis. Insets show the corresponding pre-analysis photomicrographs of the same melt inclusions.

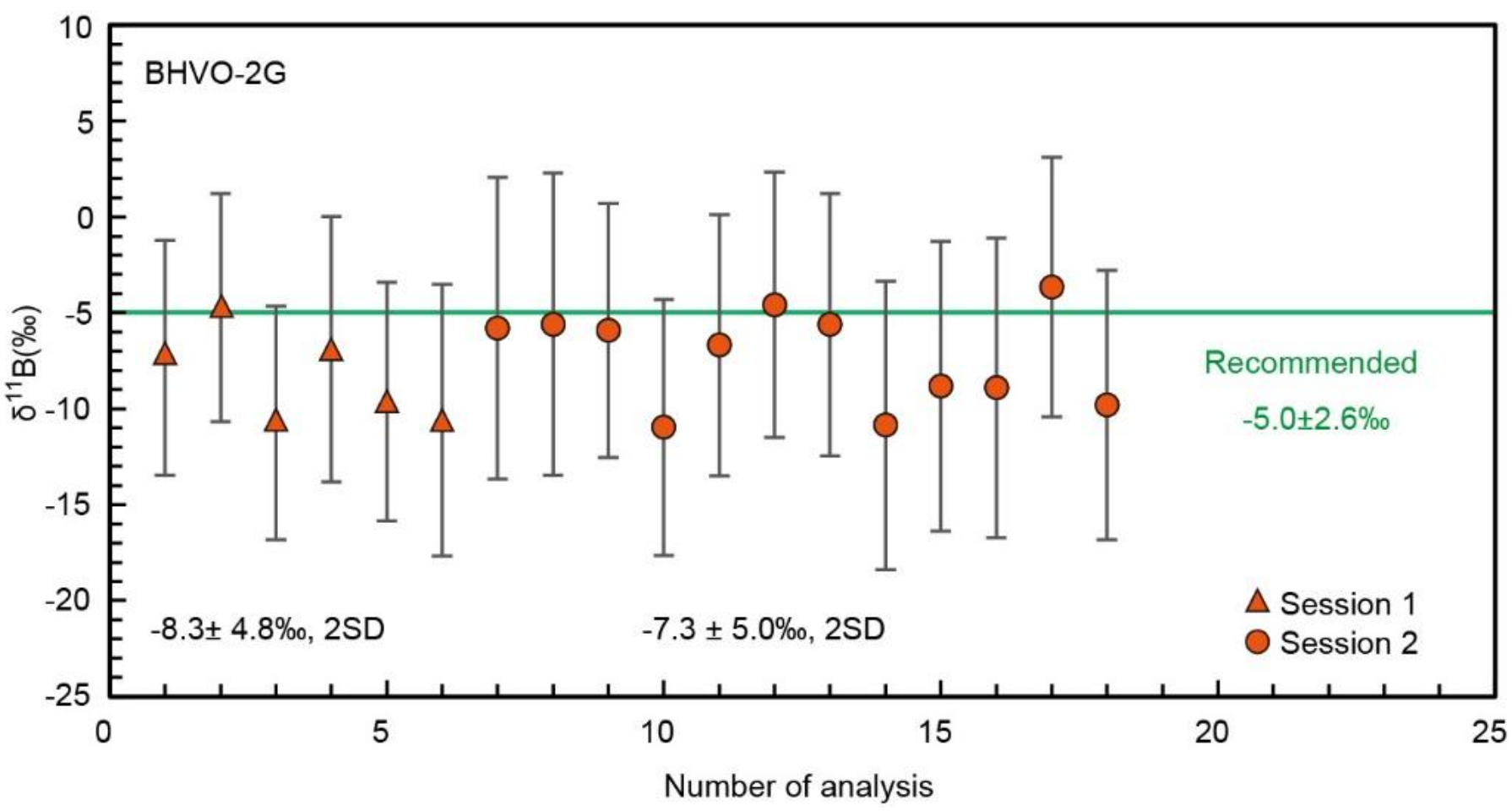

Supplementary Figure 5 | Boron isotope analyses of the international reference material BHVO-2G compared with its recommended value [10]. Error bars represent 2SE. Data supporting this figure are provided in Supplementary Data 1.

Supplementary references

1 Benton, L. D., Ryan, J. G. & Tera, F. Boron isotope systematics of slab fluids as inferred from a serpentine seamount, Mariana forearc. *Earth & Planetary Science Letters* **187**, 273-282 (2001).

2 Savov, I. P., Ryan, J. G., D'Antonio, M., Kelley, K. & Mattie, P. Geochemistry of serpentinized peridotites from the Mariana Forearc Conical Seamount, ODP Leg 125: Implications for the elemental recycling at subduction zones. *Geochemistry, Geophysics, Geosystems* **6**, doi:https://doi.org/10.1029/2004GC000777 (2005).

3 Peacock, S. M. *et al.* Thermal structure of the Costa Rica – Nicaragua subduction zone. *Physics of the Earth and Planetary Interiors* **149**, 187-200, doi:https://doi.org/10.1016/j.pepi.2004.08.030 (2005).

4 Poli, S. & Schmidt, M. W. Petrology of Subducted Slabs. *Annual Review of Earth and Planetary Sciences* **30**, 207-235, doi:https://doi.org/10.1146/annurev.earth.30.091201.140550 (2002).

5 Martin, H. & Moyen, J.-F. o. Secular changes in tonalite-trondhjemite-granodiorite composition as markers of the progressive cooling of Earth. *Geology* **30**, 319-322, doi:10.1130/0091-7613(2002)030<0319:scittg>2.0.co;2 (2002).

6 Syracuse, E. M., van Keken, P. E. & Abers, G. A. The global range of subduction zone thermal models. *Physics of the Earth and Planetary Interiors* **183**, 73-90, doi:http://dx.doi.org/10.1016/j.pepi.2010.02.004 (2010).

7 Poli, S. Carbon mobilized at shallow depths in subduction zones by carbonatitic liquids. *Nature Geoscience* **8**, 633-636, doi:10.1038/ngeo2464 (2015).

8 Chen, C., Förster, M. W., Foley, S. F. & Shcheka, S. S. Carbonate-rich crust subduction drives the deep carbon and chlorine cycles. *Nature* **620**, 576-581, doi:10.1038/s41586-023-06211-4 (2023).

9 Dasgupta, R., Hirschmann, M. M. & Withers, A. C. Deep global cycling of carbon

constrained by the solidus of anhydrous, carbonated eclogite under upper mantle conditions. *Earth and Planetary Science Letters* **227**, 73-85, doi:https://doi.org/10.1016/j.epsl.2004.08.004 (2004).
10 He, M. *et al.* Rapid determination of the original boron isotopic composition from altered basaltic glass by in situ secondary ion mass spectrometry. *Journal of Analytical Atomic Spectrometry* **35**, 238-245, doi:10.1039/C9JA00374F (2020).
