# Supplementary material for: Heavy boron isotopes reveal deep carbon recycling in hot subduction zones
Source: Nat Commun. 2026 Sep 24;17:11104. doi: 10.1038/s41467-026-78096-6 (PMC13696696; doi:10.1038/s41467-026-78096-6)
Supplement: Supplementary file 3 [file 41467_2026_78096_MOESM2_ESM.pdf]

**Description of Additional Supplementary Files**

File name: Supplementary Data 1
Description: Source data supporting all figures within the main text and Supplementary Information

File name: Supplementary Data 2
Description: Major, trace, volatile elemental, and boron isotope compositions of Panamanian and Shido-Shima melt inclusions

File name: Supplementary Data 3
Description: $CO_2$ fluxes at Southern Central American Volcanic Arc

File name: Supplementary Data 4
Description: $CO_2$ fluxes at Cascadia

File name: Supplementary Data 5
Description: Comparison of major-element compositions determined by EPMA for standards used in this study and their recommended values

File name: Supplementary Data 6
Description: Boron elemental and isotopic compositions of analyzed standards and calibration curves

File name: Supplementary Data 7
Description: Comparison of trace element concentrations determined by LA-ICP-MS for analytical standard used in this study and their recommended values

File name: Supplementary Information
Description: This file provides Supplementary figures and legends
